# Hot and Cool Executive Function in Elite- and Amateur- Adolescent Athletes From Open and Closed Skills Sports

**DOI:** 10.3389/fpsyg.2020.00694

**Published:** 2020-04-16

**Authors:** Benjamin Holfelder, Thomas Jürgen Klotzbier, Moritz Eisele, Nadja Schott

**Affiliations:** Department of Sport Psychology & Human Movement Performance, Institute of Sport- and Exercise Science, Faculty of Economic and Social Sciences, University of Stuttgart, Stuttgart, Germany

**Keywords:** 4-D multicomponent classification model, expertise, impulsivity, Game of Dice task, structural equation model, adolescents

## Abstract

**Background:**

Executive functions (EFs) not only play an important role in shaping adolescent’s goal-directed, future-oriented cognitive skills under relatively abstract, non-affective conditions (Cool EF), but also under motivationally significant, affective conditions (Hot EF). Empirical evidence suggest a link between EF, exercise and physical activity, specifically elite adult athletes appear to outperform amateur athletes in Cool EF; however, no previous studies have examined the relationship between Hot and Cool EFs and impulsivity during the developmentally sensitive period of adolescence comparing different types of sport (open- vs. closed-skills), and levels (elite athletes vs. amateurs).

**Methods:**

A total 86 boys and girls between 13 and 15 years of age (mean: 14.0, *SD*: 0.79) from different sports (track-and-field; team handball) were recruited. Participants were further divided into two groups: (a) 40 elite, and (b) 46 amateur athletes. They completed four Cool EF tasks including Trail-Making Test, Trail-Walking-Test, Flanker task, n-back-task, and one Hot EF task on Game of Dice task. Data on subjective impulsivity (UPPS Impulsive Behavior Scale; Barratt Impulsiveness Scale-15) was also collected.

**Results:**

There was a significant overall effect for expertise in favor of elite athletes (Wilks’ Lambda = 0.61, *F*(14,69) = 3.19, *p* = 0.001, $\eta_{\text{p}}^{2}$ = 0.393), but no overall main effect for type of sport or an interaction for expertise by type of sport. Specifically, elite athletes showed significantly better performances on dual tasks. For Hot EF, there were no main effects for type of sport, expertise level, training experience or training duration. We also found positive correlations among Cool EF and impulsivity measures, and between Hot EF and Impulsivity, but no significant relationship between Cool and Hot EF.

**Conclusion:**

The current understanding of the decisive cognitive abilities does not correspond to sporting reality, so that the tests frequently used are not sensitive enough to distinguish between elite and amateur athletes or different sports. However, it should also be remembered that the factors underlying complex sporting performance are multidimensional and are obviously difficult to trace back to selected partial aspects. Without being able to answer this question conclusively, we proposed a 4-D classification of experimental paradigms, in which we differentiate between tasks of different specificity, between Cool and Hot EF, and between task complexity, and type of sport.

## Introduction

While in the past the athlete’s physical ability for explaining sports performance was the main focus, various newer studies have dealt with the interaction of the athlete’s physical and cognitive performance, especially in team sports (Vestberg et al., 2012; Furley and Wood, 2016; Montuori et al., 2019). The central issue is how years of practice in different sports affect executive functions (EFs) and whether elite athletes have advantages across different EFs compared to amateur athletes (Voss et al., 2010). To answer this question, it is necessary to consider individual differences in the developmental trajectories of a person and the special features of a particular sport. If one considers the characteristics of team sports such as soccer, which requires a “cool head” for permanent decision-making under time pressure in dynamically changing and unpredictable situations (Huijgen et al., 2015), the relevance of cognitive functions becomes very clear. In this context, it should also be noted that it is not exclusively a matter of cooling the Hot EF. The crucial question is the role of Hot EF in the interaction with Cool EF and their developmental trajectories. In addition, the decision-making behavior of players, in particular toward the end of the game or in tense game situations, is additionally influenced by physical (Smits et al., 2014) and cognitive fatigue (Smith et al., 2016). Depending on the personality structure, these conditions may effect the athlete’s self-regulation (Evans et al., 2016) in terms of the motivational level, impulsive and reckless decisions, uncontrolled emotional reactions to a provocation of an opponent (e.g., the head-butt of Zinedine Zidane in the soccer world cup 2006) or violence against referees (Ackery et al., 2012). Further incidents on German football pitches show that violence against referees can no longer be dismissed as isolated actions. All these examples are related to impulsivity and self-control, which seem to pass through a sensitive phase in adolescents (Somerville and Casey, 2010; Zelazo and Carlson, 2012; Casey and Caudle, 2013). They are of great importance in all age groups, and not only in sports (Duckworth and Seligman, 2017). The overgeneralized and deliberately exaggerated statement “Adolescents have no Prefrontal Cortex” (PFC; Casey and Caudle, 2013, p. 3) – a brain area that plays a key role in brain networks underlying EF and self-control (Zelazo and Carlson, 2012; Fiske and Holmboe, 2019) – highlights this point of view.

Regarding individual performance levels, several studies showed that elite athletes outperform amateur athletes and non-athletes in EF (Vestberg et al., 2012; Jacobson and Matthaeus, 2014; Huijgen et al., 2015), but to our knowledge, no study examined both Hot and Cool EF in adolescent athletes. Based on this current state of research and the relevance described above, this study examines Hot and Cool EFs during the developmentally sensitive period of adolescence comparing different types of sport (open- vs.- closed skills), and levels of expertise (elite vs. amateur athletes).

### Hot and Cool Executive Functions: What Are They?

Self-regulation is a general ability to focus one’s attention on relevant aspects, to regulate current emotions, to suppress urgent impulses and to control behavior in a targeted and situation-adapted way (Diamond, 2013). One research strand deals mainly with the cool, cognitive aspects of self-regulation. These include cognitive psychology – with its main representative Baddeley (2000) and Baddeley et al. (2019) – and neuropsychology with Miyake et al. (2000). On the other hand, a temperament based “Hot” approach deals predominantly with the emotional-motivational aspects of self-regulation (Rothbart and Bates, 2006; Eisenberg et al., 2010; Zhou et al., 2012).

The EF’s, too, are a collective term, under which a broad spectrum of cognitive abilities are summarized, which are always needed when one wants to deviate from familiar everyday routines (Friedman and Miyake, 2017). On the one hand, this includes complex processes such as planning and monitoring the results and objectives of one’s own actions. More frequently, however, basal cognitive abilities are examined, whereby three EF are usually used, which Miyake et al. (2000) postulate: (1) Inhibition describes the ability to suppress predominant reaction tendencies; (2) Updating describes the processes of continuous monitoring and updating of working memory contents, and (3) Shifting includes the ability to change mentally between different tasks. The tripartite factor structure found by Miyake et al. (2000) in their student sample has already been reproduced several times in young adults (e.g., Ito et al., 2015), older adults (e.g., Vaughan and Giovanello, 2010), children and adolescents (e.g., Rose et al., 2012; Lee et al., 2013; Usai et al., 2013; Brydges et al., 2014).

In addition to these “Cool,” cognitive EF, Effortful Control forms the “Warm/Hot,” emotional-motivational second component of self-regulation. Effortful control is understood by Rothbart and Bates (2006) as the ability to suppress dominant responses and/or activate less dominant responses. According to this definition, the term Effortful Control should be equated with the construct of self-regulation *per se* or with EF (Diamond, 2013). Such an equation, however, does not do justice to the use of the terms in many studies. Effortful control is more often understood as self-regulation concentrated on emotions and motivations, in contrast to an executive self-regulatory component focused on cognitive processes (Zhou et al., 2012). Zelazo and Müller (2002) introduced a similar distinction between a cognitive and an affective component in 2002 – albeit in relation to EF: they described the cognitive processes required for abstract tasks as “Cool EF” and the regulation processes in affect-loaded situations as “Hot EF.” Following this terminology, the cognitive facet of self-regulation is referred to as “Cool” EF and the emotional-motivational component as “Hot” Effortful control.

EF and Effortful Control were mostly researched separately due to the different research directions. Only few studies have investigated both aspects together in a sporting context (Hongwanishkul et al., 2005; Prencipe et al., 2011; Lensing and Elsner, 2018; O’Toole et al., 2018), or they focus on special populations outside the sport context (e.g., ADHD: Geurts et al., 2006; Antonini et al., 2015; Skogli et al., 2017; Autism Spectrum Disorder: Zimmerman et al., 2016; Kouklari et al., 2017; childhood obsessive-compulsive disorder: Hybel et al., 2017). While measures of Cool EF are strongly correlated, Poon (2018) found no significant relationship between Hot and Cool EF measures in a group of 12 to 17-year-old boys and girls outside the sports context. Furthermore, from a current review there is little support for significant sex differences in Cool EF (Grissom and Reyes, 2019).

### Role of Executive Functions in Elite Sport

The distinction between open- and closed-skills sports seems to be an interesting approach for differentiated statements on the relationship between athletic performance and the performance in EF (Wang et al., 2013a; Tsai et al., 2017). Closed skills are characterized by relatively stable environmental conditions and repetitive movement patterns, as is the case with swimming or jogging. Skills that are predominantly executed in sports such as team handball, soccer, fencing or tennis, which require a constant adaptation to changing and unpredictable environmental conditions as well direct interactions with opponents, are referred to as open-skills sports (Nuri et al., 2013; Huijgen et al., 2015; Elferink-Gemser et al., 2018). Therefore, executing open skills in a functional context requires a high level of visual attention, the ability to make quick and flexible decisions, and fast movement execution (Taddei et al., 2012; Tsai et al., 2016). Thus, sports with predominantly open skills are cognitively more demanding than sports with closed skills. This leads to the assumption that athletes from sports with open skills who are constantly confronted with these cognitive stimuli should have better sport relevant cognitive performance (Voss et al., 2010; Rigoli et al., 2012; Tsai and Wang, 2015; Elferink-Gemser et al., 2018). This assumption is countered by studies in which closed-skills sports such as endurance running and cycling lead to significant improvements in EF (c.f. reviews, Sibley and Etnier, 2003; Barenberg et al., 2011; Fedewa and Ahn, 2011). For example, Wang et al. (2013a) examined inhibitory control (stop-signal task) with *n* = 60 male students (*n* = 20 tennis players, *n* = 20 swimmers, *n* = 20 sedentary controls). The tennis players achieved significantly shorter reaction times compared to swimmers and sedentary controls, while there were no significant differences between swimmers and sedentary controls. It should be noted, however, that it is methodologically difficult to clearly distinguish between open- and closed-skills sports, since in, e.g., soccer, in addition to the open skills, closed skills are also used, which underlines the importance to control for confounders like the fitness level or training hours (Wang et al., 2013b; Elferink-Gemser et al., 2018).

With regard to the current state of research on EF in sports, studies focused in particular on athletes of open-skill sports such as table tennis (Elferink-Gemser et al., 2018), ice hockey, rugby (Faubert, 2013), track-and-field (Schott and Krull, 2019), and soccer (Vestberg et al., 2012, 2017; Verburgh et al., 2014, 2016; Huijgen et al., 2015) with the focus on the comparison of Cool EF in elite and sub-elite (amateur) athletes. Although “[…] there is no hard evidence that expert athletes have superior basic cognitive abilities compared to normal, physically active controls […]” (Elferink-Gemser et al., 2018, p. 2), the aforementioned studies as well as two meta-analytic reviews (Voss et al., 2010; Scharfen and Memmert, 2019) concluded, that the level of athletic performance and the type of sport have a positive effect (with small to medium effect-sizes) on multiple subdomains of cognitive functions (Cool EF), based on non-sport specific tests.

While for Cool EF many studies confirm these findings, little is known about the significance of Hot EF in the sports context. This is the case, although Hot EF play an important role in situations, where decisions with emotional consequences need to be made and the emotional processes controlled (Kelley et al., 2015; Stein et al., 2017). Decision-making as a central feature of sports situations requires a constant control of impulses (Wang et al., 2013a), and the regulation of impulsivity (González-Hernández et al., 2019) in order to filter out the relevant information in emotionally arousing and complex situation under time pressure, and to weigh the risk of different solutions with the greatest prospect of success. On the one hand, emotions have an important influence on athletic performance (Kopp and Jekauc, 2018), but a certain level of positive emotional arousal also contributes to athletic performance (Palazzolo, 2019). On the other hand, it is known from research on decision-making that the decision-making process can be impaired in emotionally arousing situations, especially among adolescents (van Duijvenvoorde et al., 2010). Adolescence, in turn is characterized by sensitivity to impulsivity (Leshem, 2016) and challenging phases of emotion regulation (Young et al., 2019), that promote risky behavior (Hartley and Somerville, 2015), which is more pronounced in boys (Cross et al., 2011; Romer et al., 2017). Although impulsivity is multidimensional and decision-making is a complex process, there appears to be a close interaction in adolescence. In this regard, Romer (2010) and Romer et al. (2017) respectively subdivide impulsivity into the three forms *sensation seeking*, *impatience* and *acting without thinking*, the last two being related to low EF. Fino et al. (2014) also confirm the interaction between impulsivity and EF by showing that impulsivity is a predictor of EF in college students. In particular, *acting without thinking*, also known as motor impulsivity (Romer et al., 2009), seems to be important in sports, as it means a weak ability to consider different alternatives in, e.g., complex game situations or to be “mindless” at a party the night before an important competition. In addition, the relevance of *impatience* is given when a player has to decide between a fast, but risky shot at the goal or a trained tactically smart move in interaction with the teammates.

Romer et al. (2017) suggest the interaction of experience and brain development, e.g., the PFC and brain networks in this area as an important reason why adolescents react sensitively to impulsivity and tend to make more thoughtless and risky decisions. This is in accordance with the understanding of an experienced-based development of EF (Müller et al., 2013). It should be noted that many findings support the understanding of a sensitive phase in adolescents on a structural, functional and behavioral level. However, Romer et al. (2017) emphasize, that impulsivity varies from individual to individual and some conclusions drawn are the result of the stereotype “[…] the adolescent as impulsive and lacking in cognitive control” (Romer et al., 2017, p. 24).

In summary, despite the importance of Hot and Cold EF in sports, there are no studies to date that have investigated the association between Hot and Cold EF in young athletes. Therefore, the goal of this study was to investigate possible differences in Hot and Cool EF between close- skills sports (track-and-field) and open-skills sports (team handball), taking into account performance level (elite vs. amateur) and sex. Based on what we know from previous studies, we hypothesized that elite athletes would show better results than amateurs in both Cool and Hot EF. Regarding the sport type, we assume that open-skills sport athletes outperform closed-skills sport athletes in cognitive performance and that the differences between elite athletes and amateurs are less pronounced in the open-skill sports. Taking into account the findings on the sex-specific development of EF, impulsivity and risk taking behavior (Cross et al., 2011; Romer et al., 2017; Grissom and Reyes, 2019), we do not expect significant differences between girls and boys in Cool EF, but we do expect significant differences in Hot EF.

## Materials and Methods

### Participants

A total of 86 boys and girls between 13 and 15 years of age (mean age: 14.0, *SD*: 0.79) participated in the current study. Of these, 19 adolescents are elite team handball players (competing at the D-regional-squad and D-national-squad) and 27 age-matched amateur team handball players with handball experience of 3 to 13 years, practice of 1–5 days a week for 90 to 120 min. Twenty-one track-and-field athletes from the D-national-squad were included in the elite group, and another 19 age-matched subjects competed on an amateur level. The track-and-field group had an overall experience of 2 to 13 years, practice on one to five times a week for 90 to 120 min.

### Instruments

This subsection will describe the instruments used in the present study. Hot EF were assessed using the Game of Dice Task (GDT; Brand et al., 2005). Miyake et al. (2000) suggest that working memory, inhibition, and cognitive flexibility are three central aspects of EF. Based on this, we decided to assess Cool EF with a n-back task (Yun et al., 2010), a modified Flanker-task (Schott and Krull, 2019), the Trail-Making-Test (Reitan, 1958), and the Trail-Walking-Test (Schott, 2015). Two established self-report measures of impulsivity, the UPPS Impulsive Behavior Scale (Whiteside and Lynam, 2001) and the Barratt Impulsiveness Scale (BIS-15; Patton et al., 1995) were also administered.

#### Cognitive Measures

The *Game of Dice Task* (GDT; Brand et al., 2005) was used to measure decision making under risk. Participants are asked to maximize their fictive starting capital of €1000 within 18 dice throws. Subjects bet which number will be thrown by a single dice in order to maximize their starting capital. The bet can be placed on a single number or on a combination of two, three, or four numbers. Each choice is associated with fictive gains and losses depending on the probability of the occurrence of the choice: €1000 gain/loss for the choice of a single number (winning probability 1:6), €500 gain/loss for two numbers (winning probability 2:6), €200 gain/loss for three numbers (winning probability 3:6), or €100 gain/loss for four numbers (winning probability 4:6). Participants receive feedback (gain or loss) for their previous decision in a visual way, and the changed capital is shown. To analyze the risky decision making, we classified the choices of three or four number combinations as “non-risky” (winning probability of 50% and higher), and the choices of one or two numbers as “risky” (winning probability of less than 50%). We calculated a net score by subtracting the number of risky choices from the number of non-risky choices, as done in several other studies that used this task. A positive net score indicates advantageous choice behavior (range: −18 to 18).

We used the *n-back task* introduced by Yun et al. (2010). Each subject was given trials grouped in blocks, with 36 trials/block. Each trial consisted of a visual presentation of a letter for 500 ms followed by an interstimulus interval of 1500 ms. Subjects were instructed to press a target button as soon as possible for each trial in which the letter shown was the same as the letter shown n trials previously, and to press a non-target button otherwise. For the 0-back, targets were each occurrence of the letter “a.” Target responses were set to occur with 50% frequency, with accuracy computed as the number of correct responses divided by the number of trials. Subjects were given blocks of 0-back, 1-back, and 2-back. They were instructed to perform the tasks to the best of their ability, emphasizing accuracy over reaction time when possible.

Inhibition was assessed by performance during a *modified flanker task* (Eriksen and Eriksen, 1974; Schott and Krull, 2019). The stimuli were presented using E-Prime software (Psychology Software Tools, Inc., Pittsburgh, PA, United States) on a 17-in computer monitor. Responses were registered using a standard QWERTZ keyboard. Participants sat approximately 70 cm away from the screen. During the task, participants attended to a centrally presented target stimulus (Chinese letters) amid an array of laterally presented flanking stimuli. During the compatible version of the task (all signs point in to the same direction; 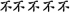), participants were required to press “L.” During the incompatible condition (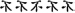, participants were required to press “S.” Five letter stimuli, measuring 4.5 cm tall and separated by 1 cm were presented for 750 ms on a white background. Participants were instructed to ignore the outside letters and to respond only to the central letter. A randomized inter-stimulus interval of 400 to 1200 ms was used, and both the number of trials within each condition and the frequency of target direction were equiprobable, with randomly presented trials within each task block. Participants were administered four blocks of 32 trials for each compatibility condition and given a brief break and encouragement between each block. For all analyses, individual trials with RT’s outside the 200–1650 ms post-stimulus onset window and incorrect trials were excluded from the RT analysis (Wu et al., 2011).

The *Trail-Making-Test* (TMT; Reitan, 1958) was used to assess EF. The reliability and validity of the TMT are well-established. The paper and pencil test consists of two parts. Part A requires the serial connection of numbers (1 to 25) randomly distributed on a white sheet of paper. Part A (TMT-A) assesses attention, visual scanning, motor speed and coordination. During part B (TMT-B), participants are asked to connect randomly positioned numbers (1 to 13) and letters (A to L) in an ascending number-letter sequence (1-A-2-B- etc.). The TMT-B assesses mental flexibility and working memory in addition to the abilities assessed by part A (Bowie and Harvey, 2006). The trials were timed using a stopwatch to the nearest 0.01 s. Due to the longer total trail length of TMT B compared to TMT A (Gaudino et al., 1995) we report the speed (cm/s) instead of the total duration. Additionally, we used a difference score (TMT-B-A) calculated by subtracting TMT-A from TMT-B. The TMT-B-A/A score is used to adjust the test time by the common motor speed element, resulting in a more accurate measure of the complex processes of cognitive flexibility and set shifting unique to TMT-B (Corrigan and Hinkeldey, 1987). These newly calculated variables represent the so-called Dual Task Effects (DTE).

To examine mobility and at the same time the use of cognitive skills such as visual scanning, vigilance, attention, and problem solving we used a dual task test. The *Trail-Walking-Test* (TWT; Schott, 2015) is based on the idea of the paper-and-pencil Trail Making Test, participants walk along a fixed pathway (TWT A), step on targets with increasing sequential numbers (i.e., 1-2-3; TWT B), and increasing sequential numbers and letters (i.e., 1-A-2-B-3-C; TWT C). Cones with numbers and/or letters are placed randomly at each of the 15 positions in a 16-m2 area (4 m × 4 m). A 30-cm diameter circle was drawn around each cone. Passage was considered to be successful when the participant stepped on the circle around the cones. The trials were timed using a stopwatch to the nearest 0.01 s following a standard procedure. The TWT was performed three times in each condition. Similar to the procedure of the TMT, the DTE were also calculated for TWT A and TWT B. To ensure consistency and to minimize errors, all trials of the TMT and TWT for all athletes were stopped by the research assistant.

#### Impulsivity Measures

The *UPPS Impulsive Behavior Scale* (Whiteside and Lynam, 2001, German version Schmidt et al., 2008) distinguishes between four dimensions of impulsivity: (1) urgency, the tendency to act rashly under conditions of negative or positive effect (12 items), (2) premeditation, the tendency to reflect on the consequences of an act before engaging in that act (11 items), (3) perseverance, an individual’s ability to remain focused on a task that may be boring or difficult (10 items) and (4) sensation seeking (12 items), the tendency to enjoy and pursue activities that are exciting and openness toward novel experiences that may be dangerous. Each item on the UPPS is rated on a four-point scale ranging from 1 (strongly agree) to 4 (strongly disagree). For each facet, higher scores indicate a higher level of impulsivity. The final questionnaire consists of 45 items and the factor structure was validated with confirmatory factor analysis. The German version of the UPPS scale in this study shows also reliable (Cronbach’s α: urgency 0.76; Premeditation 0.76; perseverance 0.77; sensation seeking 0.81) and valid acquisition of the hypothesized impulsivity facets (Kämpfe and Mitte, 2009).

The *Barratt Impulsiveness Scale-15* (BIS-15; Spinella, 2007; German version Meule et al., 2011) is a scale that has been used to assess psychological profiles and emotional regulation in risk taking sports (Cazenvae et al., 2007), and professional fighters (Banks et al., 2014). The BIS-15 is 15-item a self-report measure that is rated on a four-point scale from 1 (rarely/never) to 4 (nearly always/always), with composite scores ranging from 15 to 60. The measure assesses various aspects of impulsivity on three scales: (a) attentional impulsiveness (5 items), defined as a tendency toward quick reactions and lack of attention and cognitive control; (b) motor impulsiveness (5 items), measuring behavioral spontaneousness such as buying things spontaneously, and (c) non-planning impulsiveness (5 items), describing a lack of action planning on the level of a general attitude toward life, such as a low interest in one’s future. Moderate internal consistency has been found for the BIS-15 in this study (Cronbach’s α: non-planning impulsivity 0.67; motor impulsivity 0.67; attentional impulsivity 0.57); validity support exists with EFs (Spinella, 2007).

### Procedure

Prior to inclusion in the study, all participants signed an informed consent form. All assessments were conducted in accordance with ethical rules for research in human subjects following the Declaration of Helsinki (Edinburgh, 2000), World Medicine Association^1^. Subjects were assessed in one session; all participants started with the neuropsychological assessment, followed by the questionnaire measures. To avoid effects of the testing order within the neuropsychological assessment, two different testing orders were applied (Hot EF testing at the beginning vs. at the end). Participants were allocated randomly and counterbalanced. No significant influence of the testing order was observed. The session was conducted on a comfortable adequately illuminated room. The session had an approximate duration of 60 min. All tests were administered by a research assistant.

### Data Analysis

Statistical analyses were implemented on SPSS v.25 and AMOS 25.0 (SPSS, Chicago, IL, United States). We first explored dependent variables to examine missing data points, normality of distributions (tested by Kolmogorov–Smirnov tests), and presence of outliers.

A 2-vector scoring procedure of the flanker-task as well as the n-back-task, which uses both accuracy and reaction time, was applied on the basis of NIH Toolbox system (National Institutes of Health Toolbox Cognition Battery [NIH Toolbox CB], 2013), where each of these “vectors” ranges in value between 0 and 5. The accuracy score is calculated by 0.15625 (Flanker) or 0.1388 (n-back) points multiplied by the number of correct responses. The median reaction time values are generated using only trials with correct responses. Identical to the accuracy scores, reaction time scores range from 0 to 5 points. Because reaction time data tends to have a positively skewed distribution, a log (Base 10) transformation is be applied to each participant’s median reaction time score to create a more normal distribution of scores. The minimum RT for scoring was set to 400 ms for the Flanker and 300 ms for the n-back task, and the maximum RT for scoring is 800 ms for the Flanker task and 1300 ms for the n-back task. The following formula was used for rescaling:

$$FlankerRTscore=5-\left(5\times \left[\frac{logRT-\log\left(400\right)}{\log\left(800\right)-\log\left(400\right)}\right]\right)$$

$$n-backRTscore=5-\left(5\times \left[\frac{logRT-\log\left(300\right)}{\log\left(1300\right)-\log\left(300\right)}\right]\right)$$

After the RT scores are calculated, they are combined with the accuracy scores. If a participant’s accuracy scores are less than 80%, the final calculated score is equal to the accuracy score. However, if the accuracy score is greater than 80%, the accuracy and response time scores are combined. The calculated score combines the two vector scores and is between 0 and 10.

To determine effects of type of sport and expertise level on performance on the cognitive tasks, univariate ANCOVAs were used with sex, training experience, and training duration as covariates. Where a significant effect was found, subsequent simple contrasts (elite athletes vs. amateurs, open-skills sport vs. closed-skills sport) were performed. We used Bonferroni correction to control for multiple comparisons, resulting in corrected alpha level of 0.004 (i.e., 0.05/12).

A structural equation model was created to examine the relationship between Hot and Cool EF and impulsivity, which was calculated using AMOS 25.0. The most robust method, the maximum likelihood method, was used as the estimation model (Tabachnick and Fidell, 2013). In order to investigate the structure of the overall model and to determine the overall variance explanation contribution (R^2^), due to the large number of predictors, only those variables were included from each predictor group that could make a significant explanation contribution in their respective predictor group. Following the Tabachnick and Fidell (2013), several indices are used as model fit indices. In detail, these are the Chi-square statistics, the Tucker Lewis Index (TLI) and Bentler’s Comparative Fit Index (CFI). A statistically significant chi-square test value is to be taken as an indication of poor model fit. For the other indices, a good fit is assumed for values above 0.90 (see Hu and Bentler, 1995).

## Results

### Participants

Regarding the variables of the sports biography, we found significant differences in sporting experience between the skill groups as well as expertise level in terms of years of training [SPT: *F*(1,82) = 15.2, *p* < 0.001, $\eta_{\text{p}}^{2}$ = 0.156; E: *F*(1,82) = 10.8, *p* = 0.001, $\eta_{\text{p}}^{2}$ = 0.116] and training duration [main sport; SPT: *F*(1,82) = 5.89, *p* = 0.017, $\eta_{\text{p}}^{2}$ = 0.067; E: *F*(1,82) = 71.5, *p* < 0.001, $\eta_{\text{p}}^{2}$ = 0.466]. In addition, a significant difference between the sports type was found for training duration in additional sports [*F*(1,82) = 8.62, *p* = 0.004, $\eta_{\text{p}}^{2}$ = 0.095], which is defined as the average value (min/week) of up to three other sports. Furthermore, there was a significant difference for expertise level for BMI percentile [*F*(1,82) = 12.5, *p* < 0.001, $\eta_{\text{p}}^{2}$ = 0.132], but not for the type of sport. No individuals reported having a history of neurological problems or cardiovascular diseases, nor were any taking any medications that affect cognitive functions. Table 1 summarizes the participant’s characteristics.

**TABLE 1:** Group means (± SD) of the characteristics of the open skills sport players, and the closed skills sport athletes according to their expertise level.

	Closed-skills group (Track-and-Field)		Open-skills group (Team handball)		Statistical analysis		
	Amateur *n* = 19	Elite *n* = 21	Amateur *n* = 27	Elite *n* = 19	SPT	E	SPT x E
Age (years)	14.2 ± 0.79	13.5 ± 0.68	14.0 ± 0.81	14.1 ± 0.74	ns	ns	*
Sex	10 M, 9 F	10 M, 11 F	12 M, 15 F	11 M, 8 F	ns	ns	ns
Height (cm)	168 ± 8.57	171 ± 8.21	168 ± 7.67	170 ± 8.17	ns	ns	ns
Weight (kg)	54.9 ± 9.95	58.9 ± 10.3	54.3 ± 8.61	61.1 ± 10.3	ns	*	ns
BMI (kg/m^2^)	19.3 ± 2.43	20.0 ± 2.35	19.1 ± 1.92	21.2 ± 2.20	ns	**	ns
BMI percentile	44.5 ± 23.9	57.0 ± 23.3	43.8 ± 22.0	65.4 ± 18.8	ns	***	ns
Training experience (years)	5.11 ± 2.13	6.76 ± 2.39	7.07 ± 1.52	7.63 ± 1.57	***	***	ns
Training duration main sport (min/week)	254 ± 101	472 ± 102	228 ± 83.7	390 ± 130	*	***	ns
Training duration additional sport (min/week)	36.3 ± 67.5	65.2 ± 76.3	18.9 ± 37.3	11.1 ± 33.5	**	ns	ns

SPT, sport type; E, expertise; * p < 0.05; ** p < 0.01; *** p < 0.001.

### Cognitive Measures

#### The Game of Dice Task

Inspection of the GDT net score ranges indicated that especially athletes from track-and-field performed poorly (i.e., negative net score with a majority of disadvantageous choices). Twelve participants from closed-skills sport (30%), but only eight open-skills sport athletes (17.4%) had a negative net score on the GDT.

**FIGURE 1:**
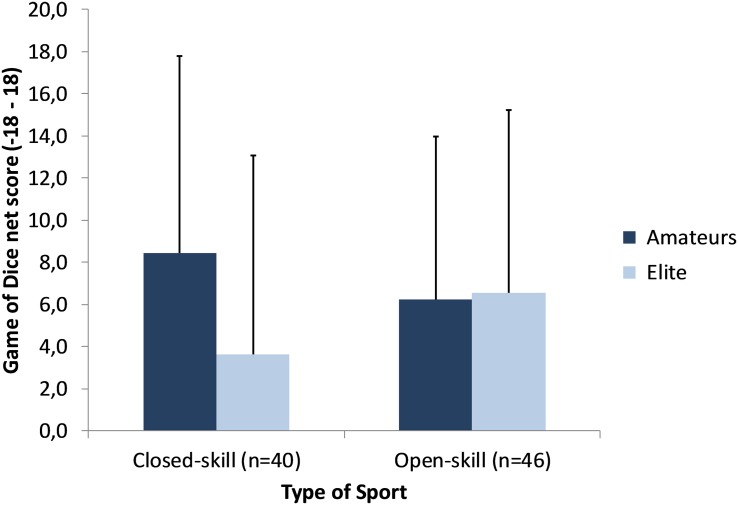
The Game of Dice Task: net differences scores (advantageous choices–disadvantageous choices; mean, SD) across amateur and elite athletes from closed- and open-skills sports.

A 2 × 2 ANCOVA computed for the GDT netscore as dependent variable and type of sport and expertise level as independent variables controlled for sex, training experience, and training duration revealed a significant main effect of sex [*F*(1,79) = 6.96, *p* = 0.010, $\eta_{\text{p}}^{2}$ = 0.081], indicating an advantageous choice behavior in girls (8.56 ± 8.09) compared to the boys (3.72 ± 8.81). A trend toward a significant interaction was found for type of sport by expertise level, [*F*(1,79) = 3.00, *p* = 0.087, $\eta_{\text{p}}^{2}$ = 0.037]. Figure 1 indicates no differences between amateurs and elite athletes from open-skills sport in contrast to a poorer performance of amateurs compared to elite athletes from closed-skills sport. There were no main effects for type of sport, expertise level, training experience or training duration.

#### N-Back Task

**FIGURE 2:**
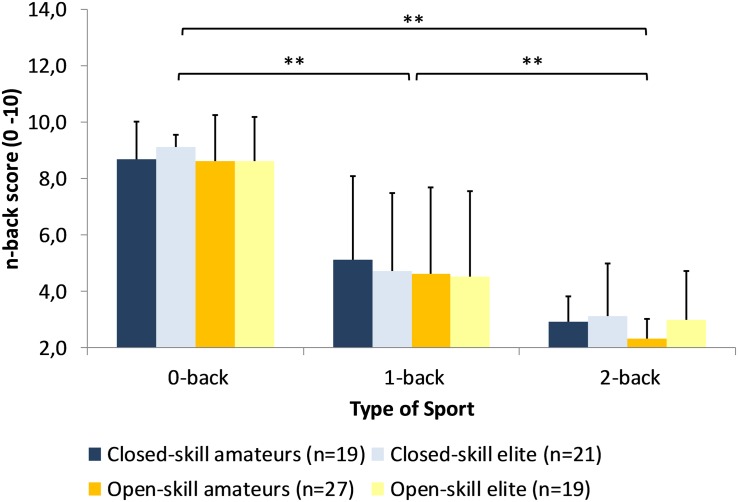
Performance of the composite score on the n-back Task as a function of expertise and type of sport (**p < 0.01).

Scores on the 0-back, 1-back, and the 2-back task are plotted in Figure 2. Repeated measures ANCOVA computed for the n-back scores as dependent variables and type of sport, expertise level as independent variables controlled for sex, training experience, and training duration revealed a significant main effect for task difficulty, [*F*(1.50,118) = 11.6, *p* < 0.001, $\eta_{\text{p}}^{2}$ = 0.128]. *Post hoc* tests demonstrated that scores were highest in the easiest task, the 0-back task (8.75 ± 1.34), and lowest for the hardest, the 2-back task (2.81 ± 1.37). Additionally, we found a significant influence of training experience on n-back task difficulty, [*F*(1.50,118) = 3.46, *p* = 0.044, $\eta_{\text{p}}^{2}$ = 0.042], indicating better working memory performance with a higher number of years of training experience. There were no effects for type of sport, expertise level, sex or training experience across the three conditions. The reaction times and accuracy for the n-back Task is presented in Supplementary Figure S1.

#### Flanker Task

**FIGURE 3:**
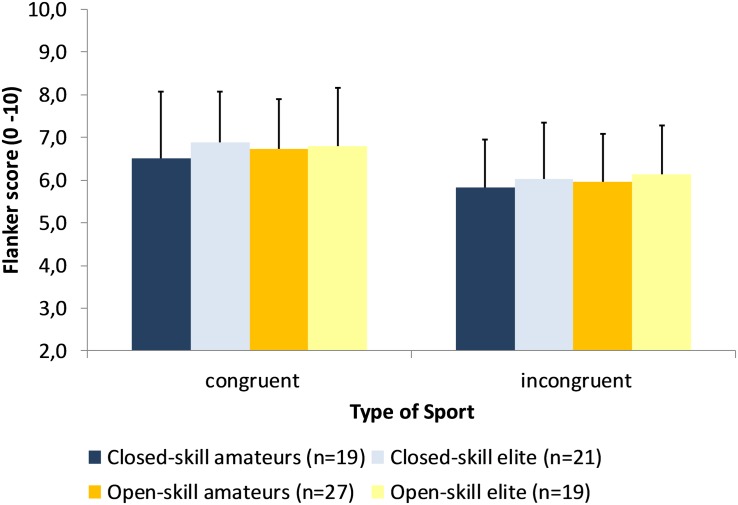
Performance of the composite score on the Flanker Task as a function of expertise and type of sport.

Scores were submitted to a 2 × 2 mixed-model ANCOVA, with condition (congruent or incongruent) as within-subjects factors, and type of sport and expertise level as between-subjects factors controlled for sex, training experience, and training duration. We found no main effects for condition, type of sport or level of expertise: athletes from the open-skills group were not faster than athletes from closed-skills group; elite athletes were not faster than amateurs (see Figure 3). No effects were found for sex, training experience, and training duration. The reaction times and accuracy for the Flanker Task is presented in Supplementary Figure S2.

#### Trail-Making-Test

**FIGURE 4:**
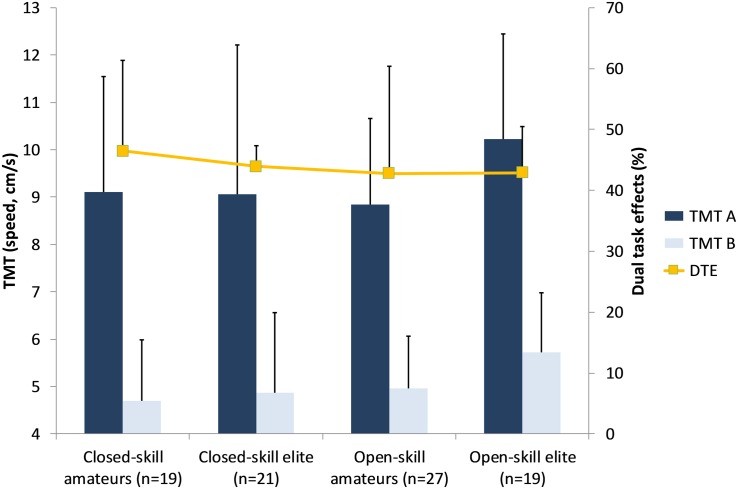
Trail-Making-Test (mean, SD), and DTEs across athletes from closed- and open-skills sports.

Repeated measures ANCOVA computed for the TMT A and TMT B times as dependent variables and type of sport, expertise level as independent variables controlled for sex, training experience, and training duration revealed a significant main effect for task difficulty, [*F*(1,79) = 8.04, *p* = 0.006, $\eta_{\text{p}}^{2}$ = 0.092] indicating longer durations on the more difficult task. *Post hoc* analysis showed a significant main effect of sex for TMT A [*F*(1,79) = 6.06, *p* = 0.016, $\eta_{\text{p}}^{2}$ = 0.071] as well as TMT B [*F*(1,79) = 6.17, *p* = 0.015, $\eta_{\text{p}}^{2}$ = 0.072], indicating better performances in girls (TMT A 9.86 ± 2.46; TMT B 5.38 ± 1.43) compared to boys (TMT A 8.66 ± 2.23; TMT B 4.71 ± 1.23). There were no main effects for expertise, type of sport, training experience, and training duration, nor were there any interaction effects for expertise level by type of sport (see Figure 4). Furthermore, no significant main effects or interactions could be found for the DTE.

#### Trail-Walking-Test

**FIGURE 5:**
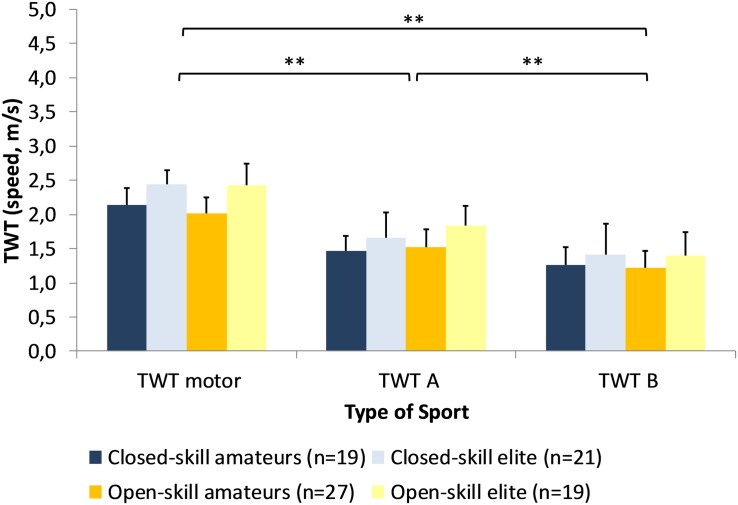
Performance of the Trail-Walking-Test (mean, SD) as a function of expertise and type of sport (^∗∗^*p* < 0.01).

Repeated measures ANCOVA computed for the TWT walking speeds as dependent variables and type of sport, expertise level as independent variables controlled for sex, training experience, and training duration revealed a significant main effect for task difficulty, [*F*(2,158) = 20.4, *p* < 0.001, $\eta_{\text{p}}^{2}$ = 0.205]. *Post hoc* tests demonstrated that speeds were highest in the easiest task, the Trail-Tracing-task task (2.24 ± 0.31 m/s), and lowest for the hardest, the TWT B task (1.32 ± 0.33 m/s) (see Figure 5). In addition, there was a significant task difficulty by sex interaction, [*F*(2,158) = 5.49, *p* = 0.005, $\eta_{\text{p}}^{2}$ = 0.065], indicating that boys outperform girls but only in the easiest condition. We also found a significant task difficulty by type of sport interaction, [*F*(2,158) = 6.60, *p* = 0.002, $\eta_{\text{p}}^{2}$ = 0.077] showing that athletes from open-skills sports outperform athletes from the closed-skills group, but only in the TWT B. Further *post hoc*-tests showed significant effects for the level of expertise with elite athletes outperforming amateurs in all three conditions (TWT motor: 2.43 ± 0.26 vs. 2.06 ± 0.25; TWT A: 1.75 ± 0.34 vs. 1.50 ± 0.24; TWT B: 1.41 ± 0.39 vs. 1.24 ± 0.25).

**FIGURE 6:**
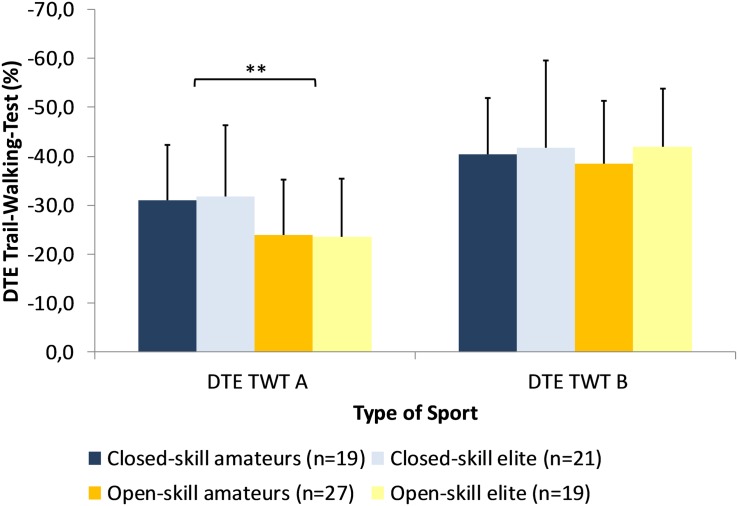
Dual Task Effects for the Trail-Walking-Test as a function of expertise and type of sport (^∗∗^*p* < 0.01).

Repeated measures ANCOVA computed for the DTEs of the TWT A and B as dependent variables and type of sport, expertise level as independent variables controlled for sex, training experience, and training duration revealed a significant main effect for task difficulty, [*F*(1,79) = 4.98, *p* = 0.028, $\eta_{\text{p}}^{2}$ = 0.059]. *Post hoc* tests demonstrated that DTEs were higher for TWT B (−40.5 ± 13.6) compared to TWT A (−27.3 ± 12.7). In addition, there was a significant interaction task difficulty x type of sport, [*F*(1,79) = 7.77, *p* = 0.007, $\eta_{\text{p}}^{2}$ = 0.090], showing similar DTEs for the TWT B (−39.9 ± 12.4 vs. −41.1 ± 14.9), but lower DTEs for the athletes from the open-skill group (−23.8 ± 11.4) compared to the closed-skill group (−31.3 ± 13.1) (see Figure 6).

### Impulsivity

Scores from the dimensions “non-planning impulsivity,” and “motor impulsivity” of the BIS-15 proved unrelated for type of sport and expertise level (see Table 2). However, an ANCOVA showed a main effect of sex with boys scoring higher than girls on the subscale attentional impulsivity (10.0 ± 2.37 vs. 9.0 ± 2.12; [*F*(1,79) = 6.32, *p* = 0.014, $\eta_{\text{p}}^{2}$ = 0.074]). Furthermore, an ANCOVA revealed a significant type of sport by expertise interaction for the Barratt Attentional subscale [*F*(1,79) = 7.85, *p* = 0.006, ${}_{\eta_{\text{p}}^{2}}$ = 0.090] with elite athletes from closed-skills sport showing higher attentional impulsivity than amateurs while elite athletes in open-skills sport exhibit lower attentional impulsivity than amateurs. The same type of sport by expertise interaction was found for the Barratt total score [*F*(1,79) = 7.78, *p* = 0.007, $\eta_{\text{p}}^{2}$ = 0.090]. Following Cohen’s interpretation of effect sizes (Tabachnick and Fidell, 2013) the effect size of group differences for the interaction was medium for attentional impulsivity and the total score.

**TABLE 2:** Group differences in impulsivity measures.

	Closed skills sport (track-and-field)		Open skills sport (team handball)		Statistical analysis			
	Amateurs *n* = 19	Elite athletes *n* = 21	Amateurs *n* = 27	Elite athletes *n* = 19	S	SPT	E	SPT x E
***BIS-15***								
Non-planning impulsivity (5–15)	11.8 ± 1.85	12.1 ± 2.30	12.7 ± 2.35	11.2 ± 3.19	ns	ns	ns	ns
Motor impulsivity (5–15)	10.6 ± 2.57	11.9 ± 2.86	11.3 ± 2.51	11.2 ± 2.23	ns	ns	ns	ns
Attentional impulsivity (5–15)	8.74 ± 2.75	10.2 ± 2.47	10.1 ± 1.92	8.84 ± 1.83	*	ns	ns	***
Total (15–60)	31.1 ± 5.16	34.1 ± 5.54	34.1 ± 4.14	31.3 ± 5.61	ns	ns	ns	***
***UPPS***								
Urgency (1–48)	26.4 ± 5.38	27.4 ± 4.15	28.5 ± 5.66	29.5 ± 4.01	ns	ns	ns	ns
Premeditation (1–44)	25.0 ± 4.43	26.7 ± 4.09	27.9 ± 4.45	26.4 ± 4.51	ns	ns	ns	ns
Perseverance (1–40)	20.1 ± 3.95	19.7 ± 3.96	22.0 ± 4.52	21.8 ± 4.75	ns	ns	ns	ns
Sensation seeking (1–48)	35.8 ± 5.18	38.5 ± 5.60	36.8 ± 6.34	38.6 ± 6.01	**	ns	ns	ns

S, sex; SPT, sport type; E, expertise; * p < 0.05; ** p < 0.01; *** p < 0.001.

2 × 2 ANCOVAs with the covariates sex, training experience, and training duration were used to detect whether means for type of sport and expertise level were significantly different on the UPPS scales. Boys scored higher on sensation seeking than girls did (39.2 ± 5.26 vs. 35.6 ± 6.00; [*F*(1,79) = 7.93, *p* = 0.006, $\eta_{\text{p}}^{2}$ = 0.091]). Elite athletes did not differ significantly from amateurs, nor were there any differences between athletes from open-skills and closed-skills sports.

### Relationship Between Impulsivity, Hot and Cool Executive Function

**FIGURE 7:**
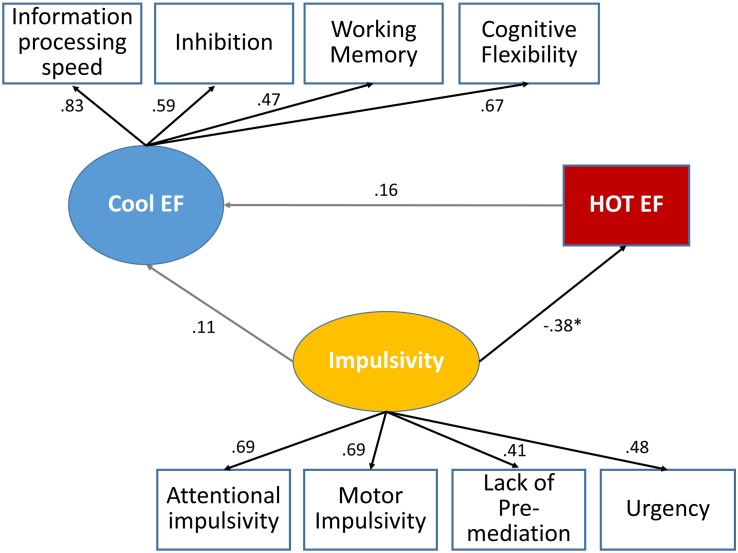
Proposed causal paths of Hot and Cool EF and Impulsivity (standardized solution) for all athletes (*n* = 86).

The structural equation model is shown in Figure 7. Standardized regression weights are shown for associations between each variable. Poor impulsivity was associated with disadvantageous choices, yet impulsivity was not correlated with Cool EF. Associations between Hot and Cool EF did not meet the significance threshold. A non-significant chi square (χ^2^ = 1.35, *p* = 0.114), the goodness-of-fit indicators (CFI = 0.932, TLI = 0.901, SRMR = 0.07, and RMSEA = 0.064) revealed that this model had a good fit, with a reasonable number of degrees of freedom (DF = 25).

## Discussion

### Discussion of Results

The purpose of this study in adolescents was to examine possible differences in Hot and Cool EF between closed-skills sport (track-and-field) and open-skills sport (team handball) athletes taking into account their performance level (elite vs. amateurs) and sex.

There are a number of studies showing that elite athletes are superior to amateurs in various perceptual cognitive tasks (e.g., Voss et al., 2010; Scharfen and Memmert, 2019), but without being able to present corresponding consistent findings to date (Elferink-Gemser et al., 2018). Regarding the Cool EF, the elite athletes in our study outperform the amateurs in all TWT conditions. Additionally, a significant influence of training experience on the working memory performance was observed, indicating better results with a higher number of training years. There are no significant differences between elite athletes and amateurs, neither for the flanker nor for the n-back task. These results are partially in line with our expectations and are not comparable to the findings of other studies in this field that compared performance in cognitive tests assessing Cool EF between expert and amateur athletes. For example, in the study by Huijgen et al. (2015), adolescent elite soccer players outperform sub-elite soccer players in inhibitory control and cognitive flexibility, but not in working memory or metacognition. The highly talented soccer players in the study by Verburgh et al. (2014) achieve better results in motor inhibition compared to the amateur soccer players, but not for visuospatial working memory, orienting and executive attention. One explanation for these inconsistent results could be that the sport-specific cognitive stimulation in sports are superimposed by other cognitive stimuli in school or recreational activities that enhance EF (Diamond and Lee, 2011; Diamond and Ling, 2016; Finch, 2019). This understanding seems particularly likely in children and adolescents, as sport is not practiced as a profession and thus competes with many other stimulants. All of this takes place at life stages in which the development of the brain is particularly dynamic (Fuhrmann et al., 2015; Nelson et al., 2016). Regardless of the type of sport, there are no significant differences between amateur and elite athletes with regard to Hot EF in the Game of Dice Task. Also for the two questionnaires investigating subjective impulsivity, no significant differences were observed for either sport type or level of expertise.

With regard to the two different types of sport, there are two significant effects for the Cool EF. One for the TWT B and the other for the DTEs in the TWT A, whereby in both cases the athletes from the open-skills sport achieved better results. These findings are in accordance with our hypothesis and the findings of previous studies in students (Wang et al., 2013a) and elderly (Tsai and Wang, 2015; Tsai et al., 2017), that athletes from open-skills sport outperform athletes from closed-skills sport. Again, the results are not as clear-cut as expected. Looking at the characteristic of the open-skills sport team handball, i.e., continuous memorization and situational application of individual and team tactical behavior in interaction with fellow players and the opposing team, cognitive skills such as mental flexibility and working memory are fundamental (Debanne et al., 2014; Furley and Wood, 2016).

For impulsivity, as measured by the UPPS Impulsive Behavior Scale no significant results were found for sports type, expertise or their interaction after controlling for covariates sex, training experience, and training duration. Apart from this, the significant type of sport by expertise interactions remain difficult to interpret for the Barratt total score and the subscale attentional impulsivity. The higher values for attentional impulsivity scores for the elite athletes compared to the amateur closed-skills-sport athletes do not confirm our hypothesis and are only partially comprehensible. It is possible that this finding can be explained by the significant differences between the type of sports due to the training duration in additional sports, with higher values for the closed-skills sport elite athletes (c.f. Table 1). It can therefore be assumed, that these athletes are confronted with more variable stimuli in different sports context, which may not result in such a clear cognitive profile. This assumption is supported by the overall higher standard deviations of the closed-skills sport athletes for the attentional impulsivity scores (c.f. Table 2). Finally, the structural equation model used to examine the interaction between Cool EF, Hot EF and impulsivity did not result in a significant relationship between Hot and Cool EF (see Figure 7).

Furthermore, no significant correlations were observed between assessments for Cool EF and Hot EF/impulsivity (see Supplementary Table S1). Thus, our findings confirm the results of Poon (2018), who found no significant relationship between Hot and Cool EF measures in a group of 12 to 17-year-old boys and girls. However, our results do not confirm the findings of Fino et al. (2014), that impulsivity is a predictor of EF. With regard to the question why there is no influence of sports type and performance level, several explanatory approaches be worth considering. First, the frequently cognitive measures used may not be specific enough to identify possible sport-related cognitive improvements, or, conversely, improved sport-specific cognitive abilities may be too specific to be transferred into cognitive measures from within the sports context (Voss et al., 2010; Faubert, 2013; Jacobson and Matthaeus, 2014). Furthermore, according to Poon (2018), the development of Cool and Hot EF during adolescence is characterized by different age-related patterns, with Hot EF developing more slowly (Prencipe et al., 2011; O’Toole et al., 2018). Finally, it can be assumed that the current understanding of the decisive cognitive abilities does not correspond to sporting reality, so that the tests frequently used up to now are not sensitive enough to distinguish between elite and amateur athletes or different sports.

A decisive question is whether a certain cognitive performance level is responsible for people performing above average in certain sports or if sport-specific training shape sport-specific cognitive profiles (chicken or egg dilemma; e.g., Voss et al., 2010; Jacobson and Matthaeus, 2014; Verburgh et al., 2014). This view is supported by the fact that even within one sport, different roles lead to or require specific cognitive profiles in order to be successful in this position (Montuori et al., 2019). The current state of research does not allow a clear answer, but it can be assumed that both perspectives are mutually dependent. In childhood, the choice of sport is often made according to what is on offer in the city or the choice depends on friends, parents, or role models (Maturo and Cunningham, 2013; Morgenroth et al., 2015). Finally, there is no doubt cognitive functions are fundamental and play a key role in sports performance, so our results addressing type of sport and expertise should be interpreted with caution.

Already Voss et al. (2010) have designated sex as an overall moderator on cognitive measures. Nevertheless, most studies in this field investigated only males (e.g., Wang et al., 2013a, b; Verburgh et al., 2014; Vestberg et al., 2017) or did not report sex-specific results (e.g., Jacobson and Matthaeus, 2014; Elferink-Gemser et al., 2018), possibly because they were not significant or meaningful. With regard to our results regarding the influence of sex on Cool EF there are a few significant differences between girls and boys, but no clear and meaningful findings were observed. These results are not only consistent with our hypothesis, but also consistent with previous studies (Vestberg et al., 2012; Grissom and Reyes, 2019).

Looking at the results of the Hot EF, girls achieved significant higher net scores in the GDT, indicating a more favorable choice behavior in girls and a more risky behavior in boys. In addition, boys scored significant higher on the BIS-15 subscale attentional impulsivity, indicating a greater propensity for rapid responses, lack of attention and cognitive control. These findings are supported by the UPPS Impulsive Behavior Scale results, which show significant higher scores for sensation seeking in boys. Therefore, the results partially confirm our hypothesis and are in accordance with previous studies (Cross et al., 2011; Romer et al., 2017).

Explanatory approaches to sex differences may be found from insights of neuroanatomical studies. Giedd and Rapoport (2010) report that sex-specific developmental trajectories for almost all structures are known. In girls, the maximum gray matter is reached 1–3 years earlier than in boys. According to Tanaka et al. (2012), brain volume and gray matter in the frontal and parietal lobes of girls develop faster than in boys, but linearly until adolescence. The cerebellar, frontal and parietal lobes are areas most often associated with EF (see systematic review of Nowrangi et al., 2014). Also in the study by Ingalhalikar et al. (2014), in which *n* = 949 children and adolescents between the ages eight and 22 years were examined, sex-specific differences are attributed to different brain sub-networks and connectivity’s. Nevertheless, it remains unclear what these results mean at the behavioral level. At the behavioral level, Brocki and Bohlin (2004) explain possible sex-specific results in EF by the fact that girls tend to be more cautious and conscientious, which is also supported by Weisberg et al. (2011).

### Discussion of Methods

One of our central questions is whether elite athletes and amateurs differ in general cognitive abilities or in certain sport-specific cognitive abilities. In taking a closer look at the current state of research, two different approaches are applied. According to Voss et al. (2010), sports training is a form of cognitive training that leads to better neuronal connectivity and plasticity and thus to improved general cognitive performance (“Cognitive Component Skill Approach”). However, this approach neglects the complex environment (specific to sports games), which also seems important to support the superiority of experts. The “Expert Performance Approach” (Mann et al., 2007; Williams et al., 2017), on the other hand, is an approach in which cognitive performance is examined in an ecologically valid and sports-specific environment and is thus representative of the specific domain of an expert. In order to be able to show clear differences between elite athletes and amateurs, sport-specific tasks with cognitive requirements that are also needed during sport are probably more suitable. Thus, the specificity of the tasks seems to play an important role in expertise research. Based on our results, a shift in the field of expertise research is necessary, which does not exclusively consider Cool EF, but instead also uses Hot EF within the “Expert Performance Approach” and the “Cognitive Component Skill Approach.”

**FIGURE 8:**
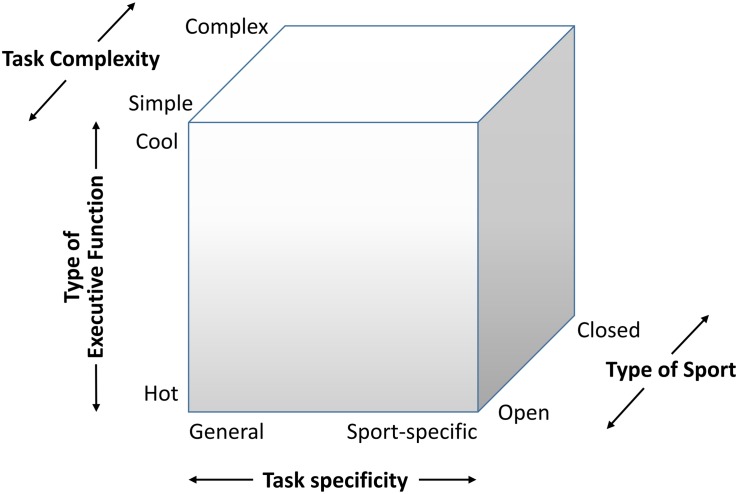
Proposed four-dimensional classification multicomponent system to examine expertise effects in sport.

In order to increase the above mentioned specificity of the tasks and to be able to observe the expertise effects more sensitively, one possibility is to think about how Cool EF tasks can be transformed into Hot EF tasks. One approach would be to select stimuli that trigger emotions. For example, faces are considered to be a special type of stimuli (Kanwisher et al., 1997) that very quickly convey emotions through different facial expressions. In addition to the appropriate selection of affective objects (e.g., faces) or situations (e.g., excessive harshness), the context in which information is received may vary depending on the relationship to persons, their personal history and other environmental conditions. Another possibility would be to generate frustration by giving false “right/wrong” feedback on the accuracy of the response. Thus, if a good performance in a reaction time experiment (e.g., flanker task) is expected from individuals, frustration may be caused by unexpected false negative feedback regarding this performance (e.g., Agnoli et al., 2019). For this transformation, however, the necessary and sufficient conditions must be defined for what qualifies a task as Hot or Cool. Assuming a continuum, each task individually has a more or less significant proportion of Hot EF requirements (Peterson and Welsh, 2014). It would be helpful to have clear criteria so that EF tasks are not randomly arranged along this Hot-Cool continuum. Due to the small number of studies dealing with this topic, there are currently no clear criteria for allocation within this continuum. In this respect, the central question remains which aspects of a task need to be manipulated to trigger certain processes (Hot or Cool processes). Without being able to answer this question conclusively, we propose a 4-Dimensional classification of experimental paradigms, in which we differentiate between tasks of different specificity (general vs. sport-specific), between Cool and Hot EF, and between task complexity (simple Cool EF: inhibition, working memory, shifting/flexibility; simple Hot EF: inhibitory control, attention control, attention shifting; complex EF: planning, problem-solving, error monitoring, updating, organizing, setting goals, creativity, cognitive regulation; complex Hot EF: delay of gratification, error monitoring, persistence, willpower, coping, resilience, emotion regulation). In addition to this three-dimensional division, we can distinguish between dominantly open (e.g., team soccer, team handball) or closed sports skills (e.g., athletics) (see Figure 8).

Such a task specificity classification makes it possible to better classify and generalize study results and to provide differentiated recommendations for training and research. It is quite conceivable that the studies with the general “Cognitive Component Skill Approach” may lead to inconsistent results, because the emotional component that arises from the respective sport is missing. Presumably, the expertise effect becomes clearer when the spectrum shifts from sport-specific Cool to Hot functions. According to this argumentation, one would expect the most significant differences in the “Expert Performance Approach” with sport-specific complex Hot EF. Moreover, this is especially the case if one can clearly distinguish between the different levels of expertise.

In this context, the concept of expertise is another important aspect to be considered. Swann et al. (2015) created a classification system of sports expertise samples that distinguishes four types of elite competitive athletes. This classification allows an operational definition of expertise in a further continuum of “eliteness” in order to be able to classify the results of studies as transparently as possible. The authors thus make an initial proposal on how a valid expert sample can be selected for future research (p. 11). Central variables of this definition within a sports comparison (highest standard of performance, success at the athlete’s highest level and experience at the athlete’s highest level) are taken into account in our sample. The variables between the sports comparisons (Competitiveness of sport in athlete’s country, global competitiveness of sport) are not taken into account since all athletes were recruited in Germany. Scharfen and Memmert (2019) also call for clarification of the terms “expert” and “elite.” According to Ericsson et al. (1993), expertise is defined by the amount of deliberate practice. The “elite” athletes are categorized as those who compete at the highest level of competition in their respective sport, which makes it possible to differentiate between competition levels. The authors use the terms high-performance athletes and low-performance athletes, so this approach is consistent with the proposal of Swann et al. (2015), who consider both aspects (experience and competition level). Scharfen and Memmert (2019) noted that a differentiation based on experience might not be sufficient to distinguish between high-performance athletes and semi-professional athletes. For future research, they recommend assigning athletes to the high performance level via the elite instead of the expert definition (p. 848).

## Conclusion

The aim of this study was to investigate possible differences in Hot and Cool EF between close- skills sports and open-skills sports, taking into account performance level and controlling for sex, training experience, and training duration. Although a significant overall effect for expertise in favor of elite athletes was observed, we did not identify main effects for type of sport or an interaction for expertise x type of sport for Cool and Hot EF. In conclusion, the results are not as clear cut as one might have been expected from previous studies. In particular with regard to methodological aspects, we see great potential to make progress in this field, which is why we proposed a 4-D classification of experimental paradigms.

As mentioned above, it is difficult to determine whether those athletes who perform better in their EF remain and establish themselves in sport, or whether better performance in their EF is due to sporting demands [nature vs. nurture problem; “which came first, the potential athlete with a particular profile of cognitive abilities, or the potential athlete that acquires a particular cognitive skill set as a result of experience-dependent learning and brain plasticity” (Voss et al., 2010, p. 823)]. It is about the source (e.g., genetic, frequencies and intensities of deliberate practice) of expertise. In this context, it is discussed whether better cognitive functions contribute to athletes becoming elite athletes or whether constant exposure in a cognitively demanding sports situation leads to better trained cognitive functions (Miyake and Friedman, 2012; Jacobson and Matthaeus, 2014; Verburgh et al., 2014; Elferink-Gemser et al., 2018). For this purpose and with regard to the performance of the EF, longitudinal studies are necessary to compare the degree of expertise. In addition, longitudinal studies could examine the influence of expertise and type of sport on the developmental trajectories of EF (both Cool and Hot) in adolescents. Previous studies of the developmental trajectories of Hot and Cool EF show that age-related improvements in Cool EF can be observed as early as childhood at the age of 8 to 13 years, while improvements in Hot EF tend to be gradual and occur later in adolescence, typically from around 14 years of age (Crone and van der Molen, 2004; Hooper et al., 2004; Prencipe et al., 2011; Poon, 2018). Poon (2018) also argues that while the Cool EF follows a linear developmental trend, the Hot EF follows a bell-shaped curve, leading to a susceptibility to risky decisions in mid-adolescence (14–15 years). Against this background, it seems necessary to use a variety of sports and comprehensive neuropsychological methods with Cool and Hot EF to cover a broad spectrum of cognitive abilities in order to investigate the relationship between expertise or sport and cognitive performance. In the recently published meta-analysis by Scharfen and Memmert (2019), the authors recommend using cognitive test methods for scouting and screening sports talents and thus optimizing their sporting development. The central question, however, is which tasks are suitable for this. Nyongesa et al. (2019) found in this context that a large number of different tasks and measures are used to assess EF in adolescents, but that the evidence of the psychometric robustness of these measures remains limited in order to prove the validity of their use in the elite context. In particular, psychometric properties are needed for the still few tasks that require Hot EF. In any case, we are convinced that Hot EFs can bring significant added value to the context of expertise research.

Furthermore, many studies mention the demand for a larger sample for higher statistical significance and more transferable statements (e.g., Huijgen et al., 2015; Elferink-Gemser et al., 2018). For future studies, it will be important to increase the statistical power of larger samples with a broad age range between childhood and young adulthood. Another central problem in expertise research is the access and willingness of competitive athletes to participate in such studies and especially in intervention studies. This would often mean taking athletes out of everyday training and, if necessary, intervening in training management. In addition, we didn’t control for confounding variables such as fitness level or personality traits. On top of the advantageous experience-based development of the elite athletes with regard to cognitive performance (Romer, 2010), possible confounders such as IQ, educational level/academic performance and socio-economic status must be taken into account (Wang et al., 2013b; Elferink-Gemser et al., 2018). This was mainly necessary in order to keep the testing effort manageable for the athletes. Furthermore, it must be clearly defined what is meant by elite. Swann et al. (2015) created a classification system of sports competence samples that distinguishes four types of elite performers. Last, but not least, we used only one task (Game of Dice Task; Brand et al., 2005) to examine Hot EF. As mentioned above, one idea is to manipulate Cool EF tasks (modified Flanker, N-back task, trail making/walking test) to transform them into tasks that increase the demand for Hot EF.

The application of experimental tasks originally developed for adults provided the opportunity to observe the development and change of cognitive functions during development (mainly Cool EF). This approach does not seem to be able to go beyond the temporary operational definitions of EF that they advocate (Miyake et al., 2000; Friedman and Miyake, 2017). Therefore, the EF construct requires further conceptual and empirical clarification, especially with regard to the exploration of emotional and motivational aspects (Hot EF). In particular, a renewed search is needed to help situate EF as a skill that is contextual and dynamically changing during development. We see EF as a consequence of the interaction between temperament and environment, and EF is strongly influenced by individual motivational drives. It seems certain that it is no longer possible to examine EF or its components from the perspective of a single methodological paradigm or from the perspective of a single theoretical position. Multidisciplinarity is crucial, which means that EF research in the next decade will almost certainly explore the dynamic relationship between brain function and structure, individual priorities, self-regulation, social context, individual information processing capacity, temperament and personal history (Müller and Kerns, 2015; Schott and Klotzbier, 2018). Such efforts are promising to demonstrate the scope of EF and the role it plays in human development, especially when it comes to the impact of expertise on cognition.

## Data Availability Statement

The datasets generated for this study are available on request to the corresponding author.

## Ethics Statement

The studies involving human participants were reviewed and approved by the University of Stuttgart. Written informed consent to participate in this study was provided by the participants’ legal guardian/next of kin.

## Author Contributions

ME and BH performed the measurements. BH, TK, and NS were involved in planning, supervising the work, processing the experimental data, performing the analysis, drafting the manuscript, and designing the figures. All authors discussed and aided in interpreting the results and worked and commented on the manuscript.

## Conflict of Interest

The authors declare that the research was conducted in the absence of any commercial or financial relationships that could be construed as a potential conflict of interest.

## Footnotes

1 http:www.wma.net

## References

[B1] Ackery A. D. Tator C. H. Snider C. (2012). Violence in canadian amateur hockey: the experience of referees in Ontario. *Clin. J. Sport Med.* 22 86–90. 10.1097/jsm.0b013e3182342b69 22252162

[B2] Agnoli S. Franchin L. Rubaltelli E. Corazza G. E. (2019). “How do you manage evaluation? attentive and affective constituents of creative performance under perceived frustration or success,” in *The Palgrave Handbook of Social Creativity Research*, Lebuda I. Glãveanu V. (Cham: Palgrave Macmillan), 225–243. 10.1007/978-3-319-95498-1_15

[B3] Antonini T. N. Becker S. P. Tamm L. Epstein J. N. (2015). Hot and cool executive functions in children with attention-deficit/hyperactivity disorder and comorbid oppositional defiant disorder. *J. Intern. Neuropsychol. Soc.* 21 584–595. 10.1017/s1355617715000752 26416095 PMC4589250

[B4] Baddeley A. D. (2000). The episodic buffer: a new component of working memory? *Trends Cogn. Sci.* 4 417–423. 10.1016/s1364-6613(00)01538-2 11058819

[B5] Baddeley A. D. Hitch G. J. Allen R. J. (2019). From short-term store to multicomponent working memory: The role of the modal model. *Mem. Cogn.* 47 575–588. 10.3758/s13421-018-0878-5 30478520

[B6] Banks S. J. Mayer B. Obuchowski N. Shin W. Lowe M. Phillips M. (2014). Impulsiveness in professional fighters. *J. Neuropsychiatr. Clin. Neurosci.* 26 44–50. 10.1176/appi.neuropsych.12070185 24515676

[B7] Barenberg J. Berse T. Dutke S. (2011). Executive functions in learning processes: do they benefit from physical activity? *Educ. Res. Rev.* 6 208–222. 10.1016/j.edurev.2011.04.002

[B8] Bowie C. R. Harvey P. D. (2006). Administration and interpretation of the trail making test. *Nat. Protoc.* 1 2277–2281. 10.1038/nprot.2006.390 17406468

[B9] Brand M. Fujiwara E. Borsutzky S. Kalbe E. Kessler J. Markowitsch H. J. (2005). Decision-making deficits of Korsakoff patients in a new gambling task with explicit rules: associations with executive functions. *Neuropsychology* 19 267–277. 10.1037/0894-4105.19.3.267 15910113

[B10] Brocki K. C. Bohlin G. (2004). Executive functions in children aged 6 to 13: a dimensional and developmental study. *Dev. Neuropsychol.* 26 571–593. 10.1207/s15326942dn2602_3 15456685

[B11] Brydges C. R. Fox A. M. Reid C. L. Anderson M. (2014). The differentiation of executive functions in middle and late childhood: a longitudinal latent-variable analysis. *Intelligence* 47 34–43. 10.1016/j.intell.2014.08.010

[B12] Casey B. Caudle K. (2013). The teenage brain: self control. *Curr. Direct. Psychol. Sci.* 22 82–87. 10.1177/0963721413480170 25284961 PMC4182916

[B13] Cazenvae N. Le Scanff C. Woodman T. (2007). Psychological profiles *and* emotional regulation characteristics of women engaged in risk-taking sports. *Anxiety Stress Coping* 20 421–435. 10.1080/10615800701330176 17999241

[B14] Corrigan J. D. Hinkeldey N. S. (1987). Relationships between parts A and B of the trail making test. *J. Clin. Psychol.* 43 402–429. 3611374 10.1002/1097-4679(198707)43:4<402::aid-jclp2270430411>3.0.co;2-e

[B15] Crone E. A. van der Molen M. W. (2004). Developmental changes in real life decision making: performance on a gambling task previously shown to depend on the ventromedial prefrontal cortex. *Dev. Neuropsychol.* 25 251–279. 10.1207/s15326942dn2503_2 15147999

[B16] Cross C. P. Copping L. T. Campbell A. (2011). Sex differences in impulsivity: a meta-analysis. *Psychol. Bull.* 137 97–130. 10.1037/a0021591 21219058

[B17] Debanne T. Angel V. Fontayne P. (2014). Decision-making during games by professional handball coaches using regulatory focus theory. *J. Appl. Sport Psychol.* 26 111–124. 10.1080/10413200.2013.801370

[B18] Diamond A. (2013). Executive functions. *Ann. Rev. Psychol.* 64 135–168. 23020641 10.1146/annurev-psych-113011-143750 PMC4084861

[B19] Diamond A. Lee K. (2011). Interventions shown to aid executive function development in children 4 to 12 years old. *Science* 333 959–964. 10.1126/science.1204529 21852486 PMC3159917

[B20] Diamond A. Ling D. S. (2016). Conclusions about interventions, programs, and approaches for improving executive functions that appear justified and those that, despite much hype, do not. *Dev. Cogn. Neurosci.* 18 34–48. 10.1016/j.dcn.2015.11.005 26749076 PMC5108631

[B21] Duckworth A. L. Seligman M. (2017). The science and practice of self-control. *Perspect. Psychol. Sci.* 12 715–718. 28862919 10.1177/1745691617690880 PMC5626575

[B22] Eisenberg N. Valiente C. Eggum N. D. (2010). Self-regulation and school readiness. *Early Educ. Dev.* 21 681–698. 10.1080/10409289.2010.497451 21234283 PMC3018834

[B23] Elferink-Gemser M. T. Faber I. R. Visscher C. Hung T. M. de Vries S. J. Nijhuis-Van der Sanden M. (2018). Higher-level cognitive functions in Dutch elite and sub-elite table tennis players. *PLoS One* 13:e0206151. 10.1371/journal.pone.0206151 30403711 PMC6221298

[B24] Ericsson K. A. Krampe R. T. Tesch-Römer C. (1993). The role of deliberate practice in the acquisition of expert performance. *Psychol. Rev.* 100:363 10.1037//0033-295X.100.3.363

[B25] Eriksen B. A. Eriksen C. W. (1974). Effects of noise letters upon the identification of a target letter in a nonsearch task. *Percept. Psychophys.* 16 143–149. 10.3758/bf03203267

[B26] Evans D. R. Boggero I. A. Segerstrom S. C. (2016). The nature of self-regulatory fatigue and “ego depletion”: lessons from physical fatigue. *Pers. Soc. Psychol. Rev.* 20 291–310. 10.1177/1088868315597841 26228914 PMC4788579

[B27] Faubert J. (2013). Professional athletes have extraordinary skills for rapidly learning complex and neutral dynamic visual scenes. *Sci. Rep.* 3:1154. 10.1038/srep01154 23378899 PMC3560394

[B28] Fedewa A. L. Ahn S. (2011). The effects of physical activity and physical fitness on children’s achievement and cognitive outcomes: a meta-analysis. *Res. Q. Exer. Sport* 82 521–535. 10.1080/02701367.2011.10599785 21957711

[B29] Finch J. E. (2019). Do schools promote executive functions? Differential working memory growth across school-year and summer months. *AERA Open* 5 1–14.

[B30] Fino E. Melogno S. Iliceto P. D’Aliesio S. Pinto M. A. Candilera G. (2014). Executive functions, impulsivity, and inhibitory control in adolescents: a structural equation model. *Adv. Cogn. Psychol.* 10 32–38. 10.5709/acp-0154-5 25157298 PMC4118776

[B31] Fiske A. Holmboe K. (2019). Neural substrates of early executive function development. *Dev. Rev. DR* 52 42–62. 10.1016/j.dr.2019.100866 31417205 PMC6686207

[B32] Friedman N. P. Miyake A. (2017). Unity and diversity of executive functions: Individual differences as a window on cognitive structure. *Cortex* 86 186–204. 10.1016/j.cortex.2016.04.023 27251123 PMC5104682

[B33] Fuhrmann D. Knoll L. J. Blakemore S. J. (2015). Adolescence as a sensitive period of brain development. *Trends Cogn. Sci.* 19 558–566. 10.1016/j.tics.2015.07.008 26419496

[B34] Furley P. Wood G. (2016). Working memory, attentional control, and expertise in sports: a review of current literature and directions for future research. *J. Appl. Res. Mem. Cogn.* 5 415–425. 10.1016/j.jarmac.2016.05.001

[B35] Gaudino E. A. Geisler M. W. Squires N. K. (1995). Construct validity in the trail making test: what makes part B harder? *J. Clin. Exp. Neuropsychol.* 17 529–535. 10.1080/01688639508405143 7593473

[B36] Geurts H. M. Van der Oord S. Crone E. A. (2006). Hot and cool aspects of cognitive control in children with ADHD: decision-making and inhibition. *J. Abnorm. Child Psychol.* 34 813–824. 10.1007/s10802-006-9059-2 17066221

[B37] Giedd J. N. Rapoport J. L. (2010). Structural MRI of pediatric brain development: what have we learned and where are we going? *Neuron* 67 728–734. 10.1016/j.neuron.2010.08.040 20826305 PMC3285464

[B38] González-Hernández J. Capilla Díaz C. Gómez-López M. (2019). Impulsiveness and cognitive patterns. understanding the perfectionistic responses in Spanish competitive junior athletes. *Front. Psychol.* 10:1605. 10.3389/fpsyg.2019.01605 31379662 PMC6646808

[B39] Grissom N. M. Reyes T. M. (2019). Let’s call the whole thing off: evaluating gender and sex differences in executive function. *Neuropsychopharmacology* 44 86–96. 10.1038/s41386-018-0179-5 30143781 PMC6235899

[B40] Hartley C. A. Somerville L. H. (2015). The neuroscience of adolescent decision-making. *Curr. Opin. Behav. Sci.* 5 108–115. 26665151 10.1016/j.cobeha.2015.09.004 PMC4671080

[B41] Hongwanishkul D. Happaney K. R. Lee W. S. C. Zelazo P. D. (2005). Assessment of hot and cool executive function in young children: age-related changes and individual differences. *Dev. Neuropsychol.* 28 617–644. 10.1207/s15326942dn2802_4 16144430

[B42] Hooper C. J. Luciana M. Conklin H. M. Yarger R. S. (2004). Adolescents’ performance on the Iowa gambling task: implications for the development of decision making and ventromedial prefrontal cortex. *Dev. Psychol.* 40:1148. 10.1037/0012-1649.40.6.1148 15535763

[B43] Hu L. T. Bentler P. M. (1995). “Evaluating model fit,” in *Structural Equation Modeling: Concepts, Issues And Application*, ed. Hoyle R. H. (Thousand Oaks, CA: Sage), 77–99.

[B44] Huijgen B. C. H. Leemhuis S. Kok N. M. Verburgh L. Oosterlaan J. Elferink-Gemser M. T. (2015). Cognitive functions in elite and sub-elite youth soccer players aged 13 to 17 years. *PLoS One* 10:e0144580. 10.1371/journal.pone.0144580 26657073 PMC4691195

[B45] Hybel K. A. Mortensen E. L. Lambek R. (2017). Cool and hot aspects of executive function in childhood obsessive-compulsive disorder. *J. Abnorm. Child Psychol.* 45:1195. 10.1007/s10802-016-0229-6 27838893

[B46] Ingalhalikar M. Smith A. Parker D. Satterthwaite T. D. Elliott M. A. Ruparel K. (2014). Sex differences in the structural connectome of the human brain. *Proc. Natl. Acad. Sci. U.S.A.* 111 823–828. 24297904 10.1073/pnas.1316909110 PMC3896179

[B47] Ito T. A. Friedman N. P. Bartholow B. D. Correll J. Loersch C. Altamirano L. J. (2015). Toward a comprehensive understanding of executive cognitive function in implicit racial bias. *J. Pers. Soc. Psychol.* 108 187–218. 10.1037/a0038557 25603372 PMC4354845

[B48] Jacobson J. Matthaeus L. (2014). Athletics and executive functioning: how athletic participation and sport type correlate with cognitive performance. *Psychol. Sport Exerc.* 15 521–527. 10.1016/j.psychsport.2014.05.005

[B49] Kämpfe N. Mitte K. (2009). A German validation of the UPPS impulsive behaviour scale: further evidence for a four-dimensional model of impulsivity. *Eur. J. Psychol. Assessm.* 25 252–259. 10.1027/1015-5759.25.4.252

[B50] Kanwisher N. McDermott J. Chun M. M. (1997). The fusiform face area: a module in human extrastriate cortex specialized for face perception. *J. Neurosci.* 17 4302–4311. 10.1523/jneurosci.17-11-04302.1997 9151747 PMC6573547

[B51] Kelley W. M. Wagner D. D. Heatherton T. F. (2015). In search of a human self-regulation system. *Annu. Rev. Neurosci.* 38 389–411. 10.1146/annurev-neuro-071013-014243 25938728 PMC4530781

[B52] Kopp A. Jekauc D. (2018). The influence of emotional intelligence on performance in competitive sports: a meta-analytical investigation. *Sports* 6:175. 10.3390/sports6040175 30551649 PMC6316207

[B53] Kouklari E.-C. Thompson T. Monks C. P. Tsermentseli S. (2017). Hot and cool executive function and its relation to theory of mind in children with and without autism spectrum disorder. *J. Cognit. Dev.* 18 399–418. 10.1080/15248372.2017.1339708

[B54] Lee K. Bull R. Ho R. M. H. (2013). Developmental changes in executive functioning. *Child Dev.* 84 1933–1953. 10.1111/cdev.12096 23550969

[B55] Lensing N. Elsner B. (2018). Development of hot and cool executive functions in middle childhood: three-year growth curves of decision making and working memory updating. *J. Exp. Child Psychol.* 173 187–204. 10.1016/j.jecp.2018.04.002 29734050

[B56] Leshem R. (2016). Brain development, impulsivity, risky decision making, and cognitive control: integrating cognitive and socioemotional processes during adolescence—An introduction to the special Issue. *Dev. Neuropsychol.* 41 1–5. 10.1080/87565641.2016.1187033 27392088

[B57] Mann D. T. Y. Williams A. M. Ward P. Janelle C. M. (2007). Perceptual cognitive expertise in sport: a meta-analysis. *J. Sport Exerc. Psychol.* 29 457–478. 10.1123/jsep.29.4.457 17968048

[B58] Maturo C. C. Cunningham S. A. (2013). Influence of friends on children’s physical activity: a review. *Am. J. Public Health* 103 e23–e38. 10.2105/ajph.2013.301366 23678914 PMC3682627

[B59] Meule A. Vögele C. Kübler A. (2011). Psychometric evaluation of the german barratt impulsiveness scale—short version (BIS-15). *Diagnostica* 57 126–133. 10.1026/0012-1924/a000042

[B60] Miyake A. Friedman N. Emerson M. Witzki A. Howerter A. Wager T. D. (2000). The unity and diversity of executive functions and their contributions to complex “frontal lobe” tasks: a latent variable analysis. *Cogn. Psychol.* 41 49–100. 10.1006/cogp.1999.0734 10945922

[B61] Miyake A. Friedman N. P. (2012). The nature and organization of individual differences in executive functions: Four general conclusions. *Curr. Direct. Psychol. Sci.* 21 8–14. 10.1177/0963721411429458 22773897 PMC3388901

[B62] Montuori S. D’Aurizio G. Foti F. Liparoti M. Lardone A. Pesoli M. (2019). Executive functioning profiles in elite volleyball athletes: preliminary results by a sport-specific task switching protocol. *Hum. Mov. Sci.* 63 73–81. 10.1016/j.humov.2018.11.011 30503984

[B63] Morgenroth T. Ryan M. K. Peters K. (2015). The motivational theory of role modeling: how role models influence role aspirants’ goals. *Rev. Gen. Psychol.* 19 465–468.

[B64] Müller U. Baker L. Yeung E. (2013). A developmental systems approach to executive function. *Adv. Child Dev. Behav.* 45 39–66. 23865112 10.1016/b978-0-12-397946-9.00003-8

[B65] Müller U. Kerns K. (2015). The development of executive function. *Handb. Child Psychol. Dev. Sci.* 2 1–53.

[B66] National Institutes of Health Toolbox Cognition Battery [NIH Toolbox CB] (2013). *Monographs of the Society for Research in Child Development.* Hoboken, NJ: Wiley. 10.1111/mono.12044 23952209

[B67] Nelson E. E. Jarcho J. M. Guyer A. E. (2016). Social re-orientation and brain development: an expanded and updated view. *Dev. Cogn. Neurosci.* 17 118–127. 10.1016/j.dcn.2015.12.008 26777136 PMC6990069

[B68] Nowrangi M. A. Lyketsos C. Rao V. Munro C. A. (2014). Systematic review of neuroimaging correlates of executive functioning: converging evidence from different clinical populations. *J Neuropsychiatry Clin. Neurosci.* 26 114–125. 10.1176/appi.neuropsych.12070176 24763759 PMC5171230

[B69] Nuri L. Shadmehr A. Ghotbi N. Attarbashi Moghadam B. (2013). Reaction time and anticipatory skill of athletes in open and closed skill-dominated sport. *Eur. J. Sport Sci.* 13 431–436. 10.1080/17461391.2012.738712 24050458

[B70] Nyongesa M. K. Ssewanyana D. Mutindi A. Chongwo E. Scerif G. Newton C. (2019). Assessing executive function in adolescence: a scoping review of existing measures and their psychometric robustness. *Front. Psychol.* 10:311. 10.3389/fpsyg.2019.00311 30881324 PMC6405510

[B71] O’Toole S. Monks C. P. Tsermentseli S. (2018). Associations between and development of cool and hot executive functions across early childhood. *Br. J. Dev. Psychol.* 36 142–148. 10.1111/bjdp.12226 29226486

[B72] Palazzolo J. (2019). Anxiety and performance. *Encephale.* 10.1016/j.encep.2019.07.008 [Epub ahead of print]. 31542211

[B73] Patton J. H. Stanford M. S. Barratt E. S. (1995). Factor structure of the barratt impulsiveness scale. *J. Clin. Psychol.* 51 768–774. 10.1002/1097-4679(199511)51:6<768::aid-jclp2270510607>3.0.co;2-1 8778124

[B74] Peterson E. Welsh M. C. (2014). “The development of hot and cool executive functions in child-hood and adolescence: are we getting warmer?,” in *Handbook of Executive Functioning*, Goldstein S. Naglieri J. (New York, NY: Springer), 45–65. 10.1007/978-1-4614-8106-5_4

[B75] Poon K. (2018). Hot and Cool executive functions in adolescence: development and contributions to important developmental outcomes. *Front. Psychol.* 8:2311. 10.3389/fpsyg.2017.02311 29367850 PMC5767838

[B76] Prencipe A. Kesek A. Cohen J. Lamm C. Lewis M. D. Zelazo P. D. (2011). Development of hot and cool executive function during the transition to adolescence. *J. Exp. Child Psychol.* 108 621–637. 10.1016/j.jecp.2010.09.008 21044790

[B77] Reitan R. M. (1958). The relationship of the trail making test to organic brain damage. *J. Consul. Clin. Psychol.* 19 393–394. 10.1037/h0044509 13263471

[B78] Rigoli D. Piek J. P. Kane R. Oosterlaan J. (2012). Motor coordination, working memory, and academic achievement in a normative adolescent sample: testing a mediation model. *Archiv. Clin. Neuropsychol.* 27 766–780. 10.1093/arclin/acs061 22777140

[B79] Romer D. (2010). Adolescent risk taking, impulsivity, and brain development: implications for prevention. *Dev. Psychobiol.* 52 263–276. 20175097 10.1002/dev.20442 PMC3445337

[B80] Romer D. Betancourt L. Giannetta J. M. Brodsky N. L. Farah M. Hurt H. (2009). Executive cognitive functions and impulsivity as correlates of risk taking and problem behavior in preadolescents. *Neuropsychologia* 47 2916–2926. 10.1016/j.neuropsychologia.2009.06.019 19560477 PMC2780004

[B81] Romer D. Reyna V. F. Satterthwaite T. D. (2017). Beyond stereotypes of adolescent risk taking: Placing the adolescent brain in developmental context. *Dev. Neurosci.* 27 19–34. 10.1016/j.dcn.2017.07.007 28777995 PMC5626621

[B82] Rose S. A. Feldman J. F. Jankowski J. J. (2012). Implications of infant cognition for executive functions at age 11. *Psychol. Sci.* 23 1345–1355. 10.1177/0956797612444902 23027882

[B83] Rothbart M. K. Bates J. E. (2006). “Temperament,” in *Handbook of Child Psychology: Social, Emotional, And Personality Development*, eds Eisenberg N. Damon W. Lerner R. M. (Hoboken, NJ: John Wiley & Sons Inc), 99–166.

[B84] Scharfen H.-E. Memmert D. (2019). Measurement of cognitive functions in experts and elite athletes: a meta-analytic review. *Appl. Cogn. Psychol.* 33 843–860. 10.1002/acp.3526

[B85] Schmidt R. E. Gay P. d’Acremont M. Van der Linden M. (2008). A German adaptation of the UPPS impulsive behavior scale: psychometric properties and factor structure. *Swiss J. Psychol.* 67 107–112. 10.1024/1421-0185.67.2.107

[B86] Schott N. (2015). Der Trail Walking Test (TWT-D): entwicklung und überprüfung der psychometrischen eigenschaften eines verfahrens zur motorisch-kognitiven interferenz bei älteren erwachsenen. *Zeitschrift für Gerontologie und Geriatrie* 48 722–733. 10.1007/s00391-015-0866-3 25801510

[B87] Schott N. Klotzbier T. (2018). “The motor-cognitive connection: indicator of future developmental success in children and adolescents?!,” in *Physical Activity and Educational Achievement: Insights From Exercise Neuroscience*, eds Bailey R. P. Meeusen R. Schäfer-Cerasari S. Tomporowski P. (London: Routledge), 111–129.

[B88] Schott N. Krull K. (2019). Stability in lifestyle behaviors – the answer to successful cognitive aging? A comparison of nuns/monks, master athletes and non-active older adults. *Front. Psychol.* 10:1347. 10.3389/fpsyg.2019.01347 31231291 PMC6567482

[B89] Sibley B. A. Etnier J. L. (2003). The relationship between physical activity and cognition in children: a meta-analysis. *Pediatr. Exer. Sci.* 15 243–256. 10.1123/pes.15.3.243

[B90] Skogli E. W. Andersen P. N. Hovik K. T. Øie M. (2017). Development of hot and cold executive function in boys and girls with ADHD: a 2-year longitudinal study. *J. Atten. Disord.* 21 305–315. 10.1177/1087054714524984 24626329

[B91] Smith M. R. Coutts A. J. Merlini M. Deprez D. Lenoir M. Marcora S. M. (2016). Mental fatigue impairs soccer-specific physical and technical performance. *Med. Sci. Sports Exerc.* 48 267–276. 10.1249/mss.0000000000000762 26312616

[B92] Smits B. L. Pepping G. J. Hettinga F. J. (2014). Pacing and decision making in sport and exercise: the roles of perception and action in the regulation of exercise intensity. *Sports Med.* 44 763–775. 10.1007/s40279-014-0163-0 24706362

[B93] Somerville L. H. Casey B. J. (2010). Developmental neurobiology of cognitive control and motivational systems. *Curr. Opin. Neurobiol.* 20 236–241. 10.1016/j.conb.2010.01.006 20167473 PMC3014528

[B94] Spinella M. (2007). Normative data and a short form of the barratt impulsiveness scale. *Intern. J. Neurosci.* 117 359–368. 10.1080/00207450600588881 17365120

[B95] Stein M. Auerswald M. Ebersbach M. (2017). Relationships between motor and executive functions and the effect of an acute coordinative intervention on executive functions in kindergartners. *Front. Psychol.* 8:859. 10.3389/fpsyg.2017.00859 28611709 PMC5447760

[B96] Swann C. Moran A. Piggott D. (2015). Defining elite athletes: issues in the study of expert performance in sport psychology. *Psychol. Sport Exerc.* 16 3–14. 10.1016/j.psychsport.2014.07.004

[B97] Tabachnick B. G. Fidell L. S. (2013). *Using Multivariate Statistics*, 6th Edn, Boston: Allyn and Bacon.

[B98] Taddei F. Bultrini A. Spinelli D. Di Russo F. (2012). Neural correlates of attentional and executive processing in middle-age fencers. *Med. Sci. Sports Exerc.* 44 1057–1066. 10.1249/mss.0b013e31824529c2 22157879

[B99] Tanaka C. Matsui M. Uematsu A. Noguchi K. Miyawaki T. (2012). Developmental trajectories of the fronto-temporal lobes from infancy to early adulthood in healthy individuals. *Dev. Neurosci.* 34 477–487. 10.1159/000345152 23257954

[B100] Tsai C. L. Pan C. Y. Chen F. C. Tseng Y. T. (2017). Open- and closed-skill exercise interventions produce different neurocognitive effects on executive functions in the elderly: a 6-month randomized, controlled trial. *Front. Aging Neurosci.* 9:294 10.3389/fnagi.2017.00294 PMC5604064 28959200

[B101] Tsai C. L. Wang C. H. Chen F. C. Pan C. Y. Huang S. Y. Tseng Y. T. (2016). The effects of different exercise types on visuospatial attention in the elderly. *Psychol. Sport Exerc.* 26 130–138. 10.1016/j.psychsport.2016.06.013

[B102] Tsai C. L. Wang W. L. (2015). Exercise-mode-related changes in task-switching perfor-mance in the elderly. *Front. Behav. Neurosci.* 9:56. 10.3389/fnbeh.2015.00056 25798097 PMC4351633

[B103] Usai M. C. Viterbori P. Traverso L. De Franchis V. (2013). Latent structure of executive function in five- and six-year-old children: a longitudinal study. *Eur. J. Dev. Psychol.* 11 447–462. 10.1080/17405629.2013.840578

[B104] van Duijvenvoorde A. C. K. Jansen B. R. J. Visser I. Huizenga H. M. (2010). Affective and cognitive decision-making in adolescents. *Dev. Neuropsychol.* 35 539–554. 10.1080/87565641.2010.494749 20721774

[B105] Vaughan L. Giovanello K. (2010). Executive function in daily life: age-related influences of executive processes on instrumental activities of daily living. *Psychol. Aging* 25 343–355. 10.1037/a0017729 20545419

[B106] Verburgh L. Scherder E. J. Van Lange P. A. Oosterlaan J. (2014). Executive functioning in highly talented soccer players. *PLoS One* 9:e91254. 10.1371/journal.pone.0091254 24632735 PMC3954684

[B107] Verburgh L. Scherder E. J. Van Lange P. A. Oosterlaan J. (2016). Do elite and amateur soccer players outperform non-athletes on neurocognitive functioning? A study among 8-12 years old children. *PLoS One* 11:e0165741. 10.1371/journal.pone.0165741 27906965 PMC5131928

[B108] Vestberg T. Gustafson R. Maurex L. Ingvar M. Petrovic P. (2012). Executive functions predict the success of top-soccer players. *PLoS One* 7:e34731. 10.1371/journal.pone.0034731 22496850 PMC3319604

[B109] Vestberg T. Reinebo G. Maurex L. Ingvar M. Petrovic P. (2017). Core executive functions are associated with success in young elite soccer players. *PLoS One* 12:e017084 10.1371/journal.pone.017084 PMC5298906 28178738

[B110] Voss M. W. Kramer A. F. Basak C. Prakash R. S. Roberts B. (2010). Are expert athletes “expert” in the cognitive laboratory? A metaanalytic review of cognition and sport expertise. *Appl. Cogn. Psychol.* 24 812–826. 10.1002/acp.1588

[B111] Wang C.-H. Chang C.-C. Liang Y.-M. Shih C.-M. Chiu W.-S. Tseng P. (2013a). Open vs. closed skill sports and the modulation of inhibitory control. *PLoS One* 8:e55773. 10.1371/journal.pone.0055773 23418458 PMC3572130

[B112] Wang C.-H. Chang C.-C. Liang Y.-M. Shih C.-M. Muggleton N. G. Juan C.-H. (2013b). Temporal preparation in athletes: a comparison of tennis players and swimmers with sedentary controls. *J. Mot. Behav.* 45 55–63. 10.1080/00222895.2012.740522 23405992

[B113] Weisberg Y. J. Deyoung C. G. Hirsh J. B. (2011). Gender differences in personality across the ten aspects of the Big Five. *Front. Psychol.* 2:178. 10.3389/fpsyg.2011.00178 21866227 PMC3149680

[B114] Whiteside S. P. Lynam D. R. (2001). The five factor model and impulsivity: using a structural model of personality to understand impulsivity. *Pers. Individ. Differ.* 30 669–689. 10.1016/s0191-8869(00)00064-7

[B115] Williams A. Fawver B. Hodges N. (2017). Using the ‘expert performance approach’ as a framework for examining and enhancing skill learning: improving understanding of how experts learn. *Front. Learn. Res.* 5 139–154. 10.14786/flr.v5i3.267

[B116] Wu M. B. Pontifex L. B. Raine L. Chaddock M. W. Voss A. F. Kramer L. (2011). Aerobic fitness and response variability in preadolescent children. *Neuropsychology* 25 333–341. 10.1037/a0022167 21443340 PMC3086950

[B117] Young K. S. Sandman C. F. Craske M. G. (2019). Positive and negative emotion regulation in adolescence: links to anxiety and depression. *Brain Sci.* 9:76. 10.3390/brainsci9040076 30934877 PMC6523365

[B118] Yun R. J. Krystal J. H. Mathalon D. H. (2010). Working memory overload: fronto-limbic interactions and effects on subsequent working memory function. *Brain Imag. Behav.* 4 96–108. 10.1007/s11682-010-9089-9 20503117 PMC2854358

[B119] Zelazo P. D. Carlson S. M. (2012). Hot and cool executive function in childhood and adolescence: development and plasticity. *Child. Dev. Perspect.* 6 354–360.

[B120] Zelazo P. D. Müller U. (2002). “Executive function in typical and atypical development,” in *Handbook of Childhood Cognitive Development*, ed. Goswami U. (Oxford: Blackwell), 445–469. 10.1002/9780470996652.ch20

[B121] Zhou Q. Chen S. H. Main A. (2012). Commonalities and differences in the research on children’s effortful control and executive function: a call for an integrated model of self-regulation. *Child Dev. Perspect.* 6 112–121. 10.1111/j.1750-8606.2011.00176.x

[B122] Zimmerman D. L. Ownsworth T. O’Donovan A. Roberts J. Gullo M. J. (2016). Independence of hot and cold executive function deficits in high-functioning adults with autism spectrum disorder. *Front. Hum. Neurosci.* 10:24. 10.3389/fnhum.2016.00024 26903836 PMC4742532

