# Supplementary material for: Hot and Cool Executive Function in Elite- and Amateur- Adolescent Athletes From Open and Closed Skills Sports
Source: Front Psychol. 2020 Apr 16;11:694. doi: 10.3389/fpsyg.2020.00694 (PMC7177013; doi:10.3389/fpsyg.2020.00694)
Supplement: Supplementary file 1 [file Data_Sheet_1.PDF]

Supplementary Material

N-back Task. Mean reaction times and accuracy are plotted in Fig. S1. Repeated measures ANCOVA computed for the n-back RTs as dependent variable and type of sport, expertise level as independent variable controlled for gender and BMI revealed no significant main effects for type of sport and level of expertise for reaction times. There was a trend towards a significant task difficulty effect [$F(1.35,108.5) = 3.10$, $p = .068$, $\eta^2 = .037$], indicating longer RTs for the more difficult conditions. There were no main effects for type of sport, expertise level, gender or BMI in each of the three conditions.

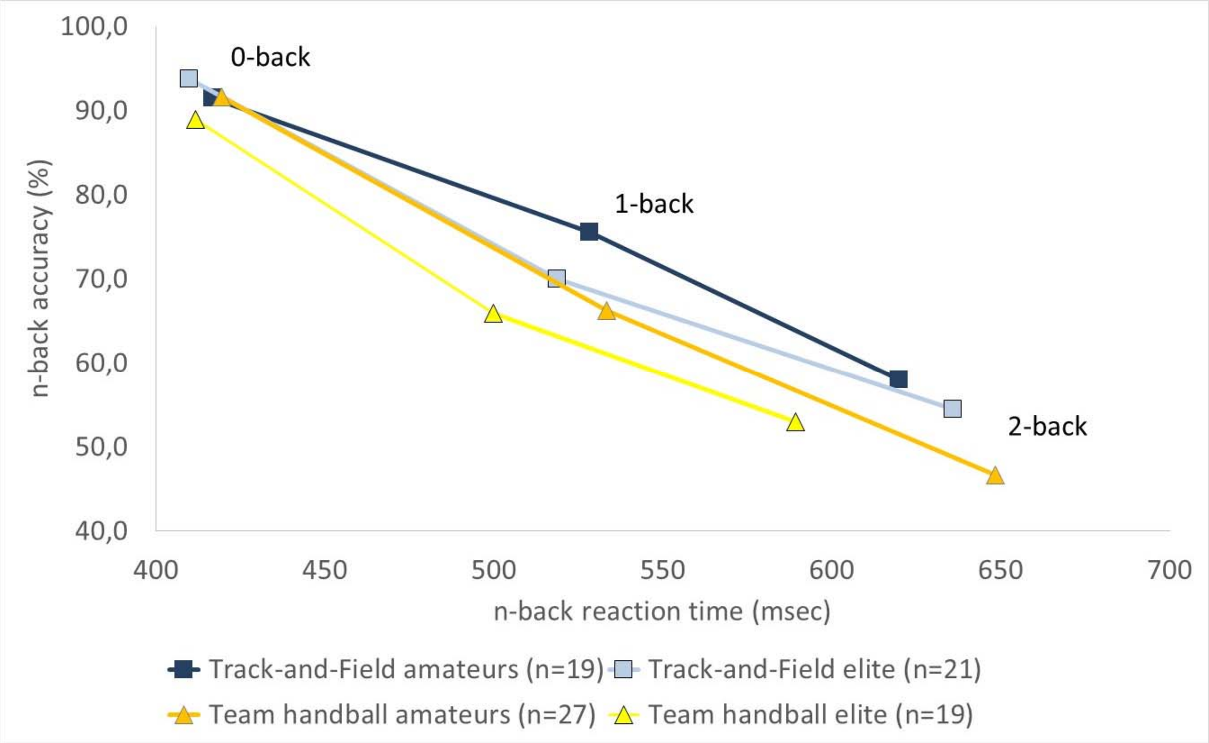

Figure S1. Reaction times and accuracy on the N-back Task as a function of expertise and type of sport

Repeated measures ANCOVA computed for the n-back accuracy rates as dependent variable and type of sport, expertise level as independent variable controlled for gender and BMI revealed a significant task difficulty effect [$F(1.82,145) = 9.38$, $p < .001$, $\eta^2 = .105$], indicating lower accuracy rates for the more difficult conditions. There were no main effects for type of sport, expertise level, gender or BMI in each of the three conditions.

Flanker task. RTs were submitted to a 2 × 2 mixed-model ANCOVA, with condition (congruent or incongruent as within-subjects factors, and type of sport and expertise level as a between-subjects factor controlled for gender and BMI. We found a significant main effect of condition [$F(1,80) = 11.3$, $p = .001$, $\eta^2=.124$], with congruent trials (560 ± 48.9) faster than incongruent trials (619 ± 58.3). There was no main effect of type of sport or level of expertise: Team handball players were not faster than athletes from track-and-field; experts were not faster than novices (see figure S2). No effects were found for gender or BMI. Accuracy rates were submitted to the same ANOVA as RTs. No main effects or interactions were significant (all ps > .1).

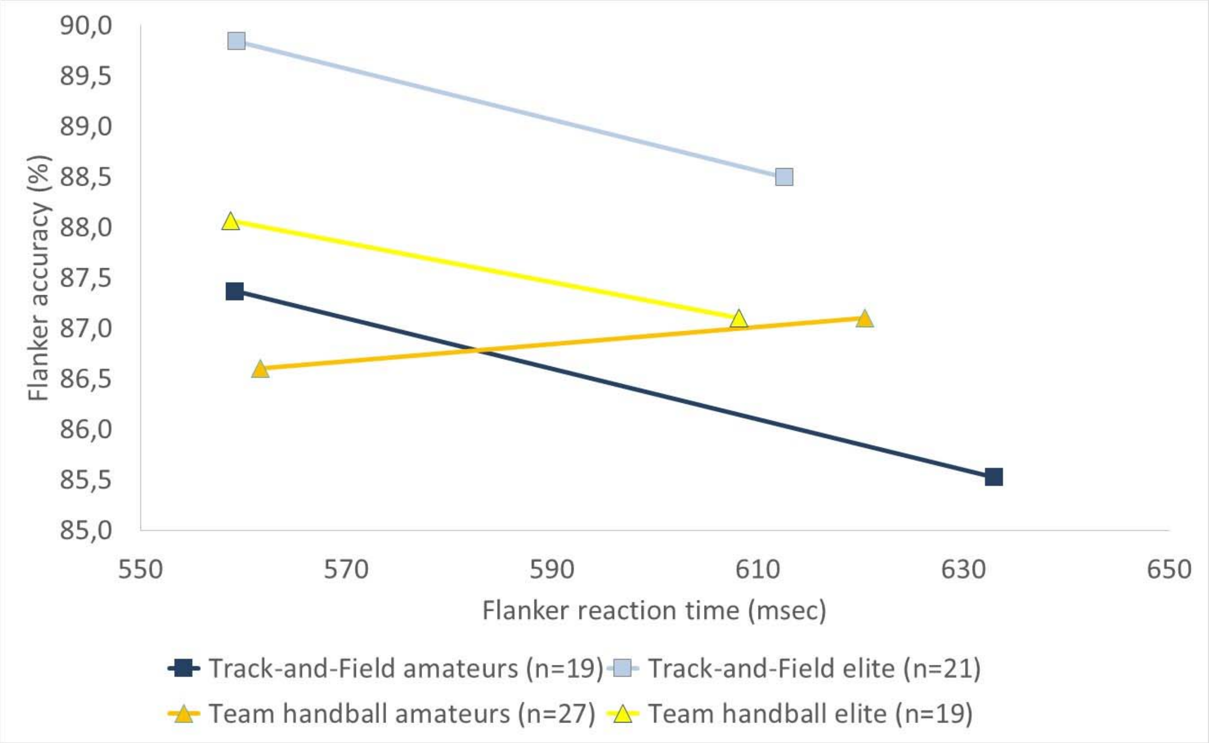

Figure S2. Performance of reaction times and accuracy on the Flanker Task as a function of expertise and type of sport (values on the left side: congruent condition; values on the right side: incongruent condition)

Relationship between Hot and Cool Executive Function and Impulsivity

Table S1. Correlations between Hot and Cool Executive Function and Impulsivity

	(2)	(3)	(4)	(5)	(6)	(7)	(8)	(9)	(10)	(11)	(12)
(1) Information processing speed	.485**	.329**	.600**	.032	.038	.025	-.094	-.068	.035	.184	.099
(2) Inhibition		.496**	.290**	.131	.088	.105	.091	.006	.053	.155	.121
(3) Working memory			.287**	.052	-.032	-.053	.011	-.007	-.024	.074	.117
(4) Cognitive Flexibility				.192	-.153	-.025	-.005	-.150	-.075	.117	.009
(5) GOD net score					-.294**	-.231*	-.068	-.051	-.233	-.128	-.125
(6) attentional impulsivity						.463**	.194	.222*	.201	.174	.396**
(7) motor impulsivity							.099	.020	.372**	.323**	.301
(8) non-planning impulsivity								.231*	.271*	-.070	.073
(9) Lack of Perserverance									.392**	-.263*	.339**
(10) Lack of Premediation										.051	.128
(11) Sensation seeking											-.043
(12) Urgency											

Note. Information processing speed (TMT A, TWT A, 0-back); Working memory (1-back, 2-back); Inhibition (Flanker congruent, incongruent); Cognitive flexibility (TMT B, TWT B)
